# Oxidation of Flavin by Molecular Oxygen: Computational Insights into a Possible Radical Mechanism

**DOI:** 10.1021/acsomega.4c00307

**Published:** 2024-05-22

**Authors:** Jernej Stare

**Affiliations:** National Institute of Chemistry,Hajdrihova 19, SI-1000 Ljubljana, Slovenia

## Abstract

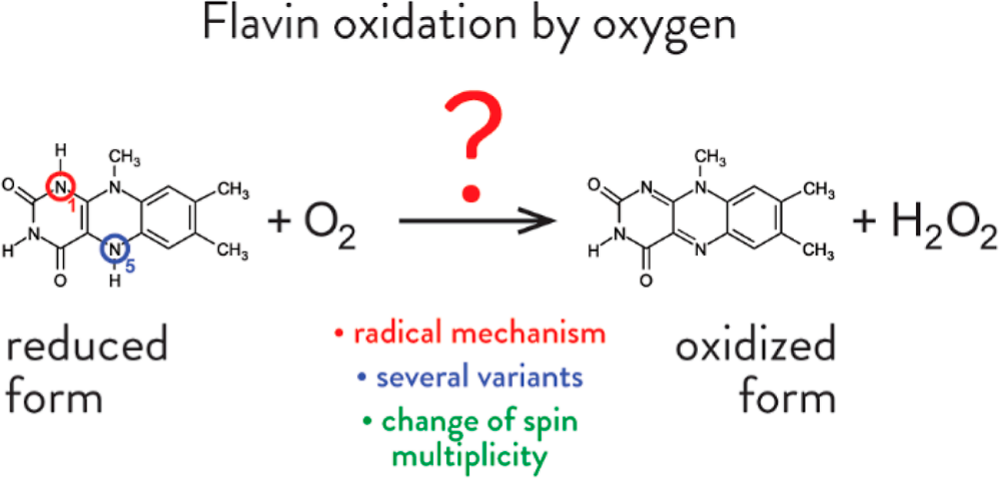

As a highly electrophilic moiety capable of oxidizing a variety of small organic molecules and biomolecules, flavin is an important prosthetic group in many enzymes. Upon oxidation of the substrate, flavin is converted into its reduced (dihydrogenated) form. The catalytic cycle is completed through oxidation back to the oxidized form, thus restoring the enzyme’s oxidizing capability. While it has been firmly established that oxidation of the reduced form of flavin is cast by molecular oxygen, yielding oxidized flavin and hydrogen peroxide, the mechanism of this process is still poorly understood. Herein, we investigate the radical mechanism, which is one of the possible reaction mechanisms, by quantum chemical calculations. Because molecular oxygen exists as a triplet in its electronic ground state, whereas the products are singlets, the reaction is accompanied by hopping between electronic surfaces. We find that the rate-limiting factor of flavin oxidation is likely associated with the change in the spin state of the system. By considering several possible reactions involving flavin and its derivatives in the radical form and by examining the corresponding parts of the potential energy surface in various spin states, we estimate the effective barrier of the kinetically and thermodynamically preferred variant of flavin oxidation to be about 15 kcal/mol in the gas phase and about 7 kcal/mol in a polar (aqueous) environment. This is in agreement with kinetic studies of the corresponding monoamine oxidase enzymes, confirming the radical mechanism as a viable option for flavin regeneration in enzymes.

## 1 Introduction

Flavin chemistry plays an important part in life processes. Enzymes using flavin as a cofactor regulate a range of reactions, encompassing a class of enzymes named flavoenzymes consisting of hundreds of proteins. For example, human genome encodes 90 flavoenzymes, of which a majority (84%) utilize flavin adenine dinucleotide (FAD) as a cofactor, whereas a minor part (16%) require flavin mononucleotide (FMN); five human flavoenzymes include both FAD and FMN.^1^ Most of the flavoenzymes (>90%) catalyze redox transformations (oxidoreductases), but to a minor extent, they perform other functions, e.g., examples of flavin-based transferases, lyases, isomerases, and ligases are known.^2^ In addition, flavin serves as a signaling and sensing molecule in diverse biological processes. Interestingly, as much as 60% of human flavoproteins are associated with diseases and disorders caused by mutations in their pertinent genes.^1^ Among many universal, long known examples of flavin-dependent enzymes is succinate dehydrogenase involved in oxidative conversion of succinate to fumarate in the citric acid cycle, which uses FAD prosthetic group as the oxidizing agent.^3^ Another important example of flavoenzymes is monoamine oxidases (MAOs) controlling levels of monoaminergic neurotransmitters in the central nervous system. MAO enzymes use covalently bound FAD prosthetic group facilitating oxidative decomposition of neurotransmitters such as dopamine and serotonin.^4−6^ Other examples of flavin-dependent enzymes include d-amino acid oxidase regulating the levels of nonacidic amino acids in tissues by oxidative deamination,^7^ ferredoxin-NADP^+^ reductase involved in the last stage of electron transfer chain in photosynthesis,^8^ acyl-CoA dehydrogenases facilitating metabolism of fatty acids in mitochondria by catalyzing β-oxidation of fatty acids using an FAD cofactor,^9^ and many others. Flavin is also involved in the nitrogen fixation process.^10^ Among flavoenzymes featuring important bioanalytical application is glucose oxidase used in blood glucose biosensors;^11^ this enzyme uses FAD in the glucose oxidation process.^12^ Worthy to note, flavins are photosensitive and can undergo reactions mediated by light. This feature is utilized by light-sensitive proteins. For example, the function of phototropins (blue light receptors in plants) involves a light-induced formation of a covalent adduct between a conserved cysteine and the FMN cofactor.^13−15^ On the other side, flavin-mediated reactions are also capable of emitting light, a feature exploited by certain bacterial luciferase enzymes in which the reduced form of FMN (FMNH_2_) is oxidized by molecular oxygen in the presence of long-chain aliphatic aldehyde to the hydroxyflavin intermediate in an excited electronic state; further transformation to the oxidized FMN product results in light emission.^16^

**Figure 1:**
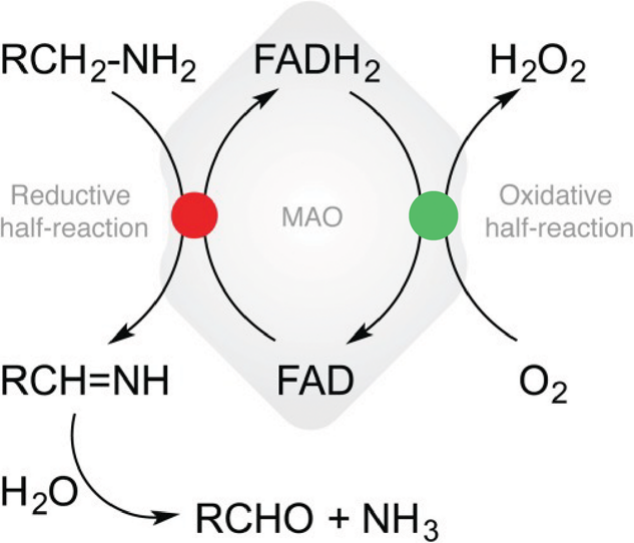
Catalytic cycle of monoamine oxidase (MAO) enzymes as an example of the oxidoreductive processes of flavin-containing entities in enzymatic reactions. The red dot indicates the catalytic step, whereas the green dot denotes flavin oxidation restoring the catalytic function of MAO. Reproduced from [Repic, M.; Vianello, R.; Purg, M.; Duarte, F.; Bauer, P.; Kamerlin, S. C. L.; Mavri, J., Empirical Valence Bond (EVB) simulations of the hydride-transfer step in the monoamine oxidase B-catalyzed metabolism of dopamine. Proteins 2014, 82 (12), 3347–3355].^18^ Copyright 2014 John Wiley and Sons.

Focusing on the most common function of flavoenzymes, that is, catalyzing oxidoreductive processes, catalytic cycles of these processes include interconversion between the oxidized and reduced form of flavin, e.g., in FAD-mediated reactions, this includes FAD and FADH_2_ entity as the oxidized and reduced species, respectively. When involved in oxidation of the enzyme’s substrate, flavin is reduced in the chemical step (reductive half-reaction as experienced by flavin) and subsequently regenerated in the other part of the catalytic cycle (oxidative half-reaction). An example of such a cycle is shown in Figure 1. Since a number of chemical transformations are mediated by flavoenzymes, a variety of diverse substrates (other than those displayed in Figure 1) can be involved in the chemical step. On the other hand, regeneration of flavin back to its oxidized form is normally cast by molecular oxygen (O_2_), releasing hydrogen peroxide (H_2_O_2_) as the side product.^17^ That said, mechanistic and kinetic aspects of the latter reaction have proven to be far from trivial, which will be further explained below.

Enzyme regeneration comprises an essential part of the catalytic cycle, and depending on the pertinent free energy barrier, its kinetics may be relevant for the catalytic efficiency of the enzyme. When regeneration proceeds at higher rate than the chemical step (i.e., when it features lower free energy barrier), the kinetics of regeneration does not interfere with the chemical step. However, if the barrier associated with enzyme regeneration is comparable to or higher than that of the chemical step, the regeneration mechanism may control the enzyme kinetics, calling for in-depth investigation. Indeed, for oxidative regeneration of flavin, evidence exists that the pertinent barrier may be, at least in certain cases, high enough to control the kinetics of the catalytic cycle. Earlier studies of the catalytic mechanism of monoamine oxidase B (MAO-B) suggest that oxidation of a free reduced enzyme with oxygen represents the rate-limiting step.^6^ Another kinetic study of MAO-B implies that the overall rate is determined by a complex function of both the reductive and oxidative half reactions.^19^ This implies that the oxidation barrier is of a comparable magnitude to the one of the chemical step—that is, about 15–18 kcal/mol, as measured for a variety of substrates of both variants of MAO.^20−23^ In contrast to MAO-B, the structurally and functionally similar monoamine oxidase A (MAO-A) features the chemical step as rate-limiting, and enzyme/flavin oxidation proceeds at significantly higher rates than the catalytic step.^24^ This suggests that the flavin oxidation barrier can vary even between very similar enzymatic environments, let alone among the whole variety of flavoenzymes.

**Figure 2:**
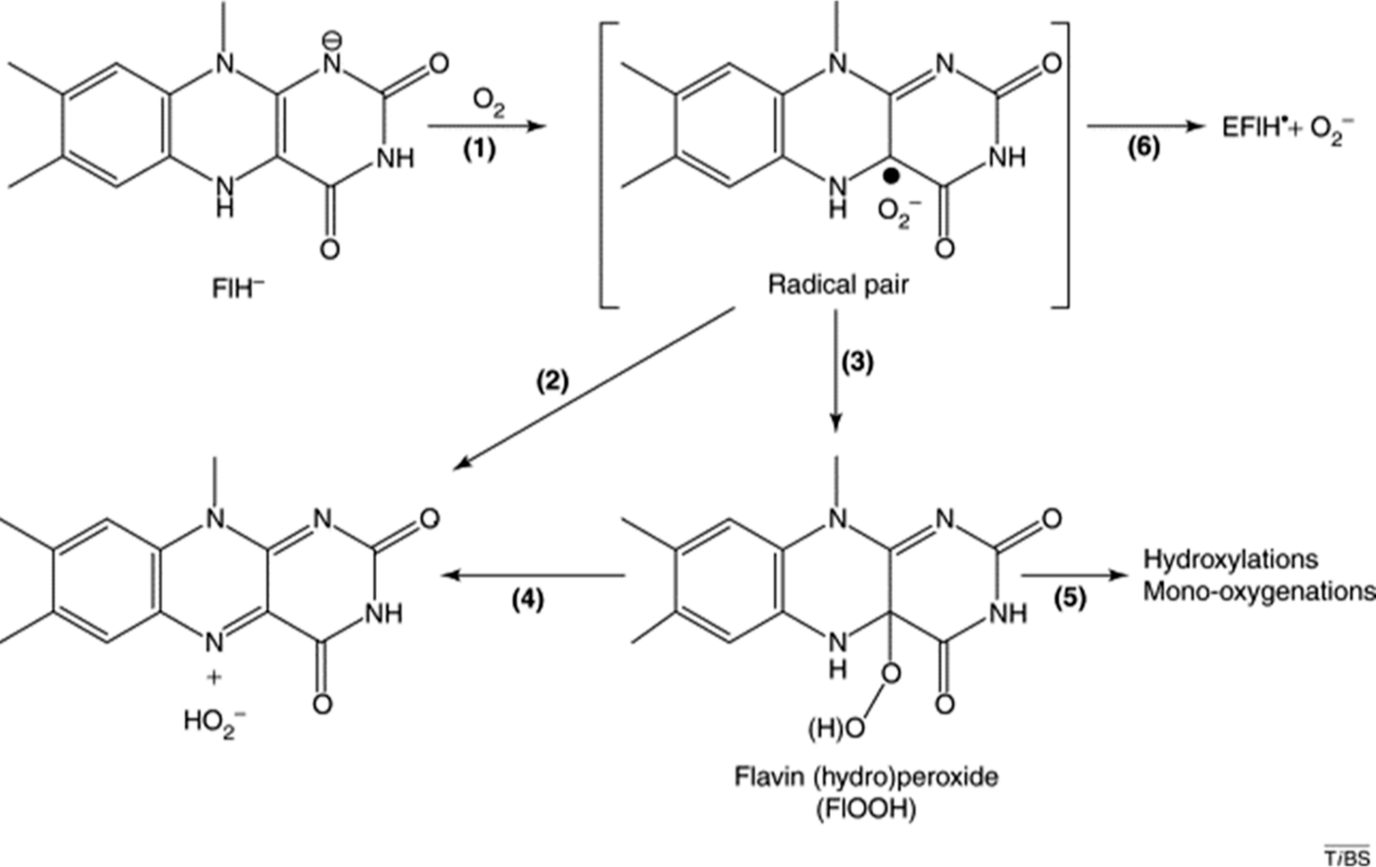
Proposed mechanism of flavin oxidation in enzymes. Note that the scheme uses notation different from the one in the present work, e.g., “Fl” stands for (oxidized) flavin, essentially equivalent to the presently used notation “LFN” corresponding to (oxidized) lumiflavin (see below). Reprinted from [Chaiyen, P.; Fraaije, M. W.; Mattevi, A., the enigmatic reaction of flavins with oxygen. Trends Biochem Sci 2012, 37 (9), 373–380].^17^ Copyright 2012, with permission from Elsevier.

Despite the simplicity implied by Figure 1, a detailed mechanism of flavin oxidation either in solution or in an enzymatic environment is elusive. In enzymes, this reaction exhibits extraordinary versatility that is unmatched among organic cofactors.^17^ Kinetics of flavin oxidation in enzymes spans several orders of magnitude, and no correlations facilitating prediction of the reaction mechanism and its kinetics from the structural parameters could have been established.^25^ Among examples demonstrating complexity of flavin oxidation in enzymes, kinetic evidence suggests that the FAD prosthetic group in MAO enzymes is regenerated by O_2_ more rapidly in the presence of the substrate in the active site, i.e., involving a ternary complex of the enzyme, substrate, and O_2_.^6,24^ A similar mechanism of flavin oxidation based on a ternary complex has also been observed in flavin-dependent luciferases.^16^ While the mechanism of flavin oxidation appears to be pronouncedly case-dependent, it is generally assumed that it involves single electron transfer from flavin to the oxygen molecule, resulting in a caged radical pair; depending on the nature of the enzyme, oxidation further proceeds either directly to the oxidized flavin molecule or via the covalent flavin hydroperoxide intermediate (Figure 2).^17^

The importance and versatility of biochemical reactions involving flavin call for profound examination of the underlying physical background by accurate computational treatments. The structure of flavin, its chemistry, electronic states, and spectroscopy have been subject of extensive research, employing classical, quantum, and multiscale treatments.^26^ However, research attempts in elucidating flavin oxidation by O_2_ appear to be scarce, e.g., DFT studies confirm that in MAO enzymes, lysine residues interacting with the N5 atom of flavin ring via water-bridged hydrogen bonds appear to be important for the flavin reaction with O_2_.^27,28^

To the best of our knowledge, flavin oxidation by O_2_ has not yet been the subject of a detailed investigation based on quantum chemistry protocols suitable for chemical reactivity. Therefore, the scope of the present study is to provide insights into selected aspects of flavin oxidation by molecular oxygen. Herein, we scrutinize the radical mechanism by using the established DFT methodology and examine whether its estimated kinetics supports the experimental observation suggesting that the free energy barrier in an enzyme environment is in the range of ∼15 kcal/mol, as appears to be the case in the MAO-B enzyme.^19^ The choice of MAO-B as a relevant reference case is supported by the fact that the catalytic performance of MAO enzymes extends to psychiatry and neurology because MAO enzymes are involved in metabolism of monoaminergic neurotransmitters in the central nervous system, and deviation from their normal performance is related to various neurological pathologies.^29−33^ In addition, the oxidative regeneration of flavin is a source of oxidative stress due to the release of hydrogen peroxide, which may further produce a variety of reactive oxygen species, causing damage to cells and tissues. While these effects are probably common to many flavoenzymes, they are particularly pronounced in the central nervous system, where the oxidative stress associated with the action of MAO enzymes may lead to neurodegeneration and to associated diseases (Alzheimer and Parkinson).^34−36^ Therefore, MAO enzymes are popular targets for research of novel inhibitors^37^ and extensive mechanistic studies.^4,38−40^ In any case, the molecular mechanism of flavin oxidation in biomolecular systems tackles the issue of cell damage caused by oxidative stress, which is an intriguing problem on its own.

Herein, we limit our focus to selected aspects of the radical mechanism. Our choice derives from several factors. First, the fact that molecular oxygen (O_2_) is a biradical (spin triplet) in its electronic ground state, whereas the final products are evidently spin-paired (singlets), requires that the change of spin (related to the crossing between the, respective, potential energy surfaces) occurs during the reaction, which suggests that radical entities may be involved in the mechanism. Second, our preliminary computational assessment suggests that the potential energy landscape for the flavin–oxygen reacting system is extremely complex, with stationary points (particularly transition states) difficult to be properly estimated. We found that the complexity issue is particularly pronounced with the possible concerted polar reaction mechanism, whereas the relative simplicity of the radical mechanism, which can be broken down to simple steps, allows for a more reliable computational characterization. Third, the reduced form of flavin is structurally (and possibly chemically) similar to anthrahydroquinone for which the radical oxidation mechanism by O_2_ has been firmly established, also by computational studies (the anthraquinone process is an important pathway for industrial production of H_2_O_2_).^41^ Consequently, in an attempt to derive concise (yet not necessarily complete) characterization of the flavin oxidation process, this work focuses on the hypothetical radical mechanism.

**Figure 3:**
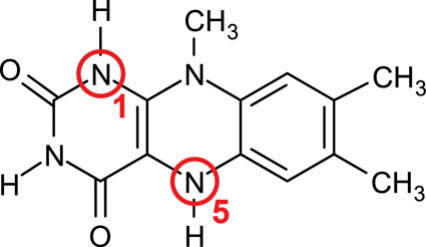
Reduced form of lumiflavin (LFNH_2_ henceforth, see Figure 4) with indicated nitrogen atoms at sites 1 and 5. In the oxidation process, both hydrogens bound to these nitrogen atoms are abstracted, yielding the oxidized form (LFN, see Figure 4).

The most common entity found in flavoenzymes is FAD. For the sake of computational cost reduction, we use in this study a truncated system lumiflavin (LFN), from which the adenine nucleotide and ribitol entities have been removed and substituted by a methyl group, significantly reducing the model size (Figure 3). The triple-ring isoalloxazine moiety common to all flavins and crucial for their chemistry is retained. In this article, LFN denotes the oxidized form, whereas for the reduced form of LFN, the LFNH_2_ acronym will be used. The structure of LFNH_2_ is displayed in Figure 3.

The two hydrogens subject to removal in the course of the reaction are bound to ring nitrogen atoms at positions 1 and 5 (Figure 3); the product is the LFN molecule with no hydrogens bound to both N1 and N5. For the assumed radical mechanism, the net reaction
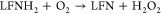
is broken down to several steps in which one or more of the involved molecular entities are present in a radical form—that is, their electronic structure features an unpaired electron. The radical mechanism studied herein includes the following steps:
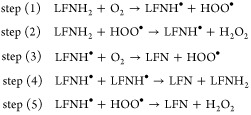


Of those, step (1) represents initialization (production of semioxidized flavin, LFNH^•^, and hydroperoxyl radicals, HOO^•^ out of the reactants); steps (2) and (3) can be regarded as propagation (further production of radicals by means of the already formed ones); whereas steps (4) and (5) are associated with termination in which radicals are combined to form spin-paired products. Among the above steps, steps (3–5) yield the main product, which is the oxidized form of LFN. Thus, the net reaction may proceed along various pathways which we scrutinize in the present study, but all the pathways include step (1) as the initial source of radicals required by further steps. In the present work, each of these steps has been characterized by computation of the structure and energy of reactants, products, and the transition state. Then, considering the net reaction as various possible combinations of these steps, one can deduce the most plausible mechanism on the basis of the lowest-energy pathway. While inclusion of the explicit enzymatic environment such as that of MAO-B is beyond the scope of this study, effects of the polar medium have been estimated by the self-consistent reaction field (SCRF) implicit solvation methodology available in quantum chemistry program packages.

**Figure 4:**
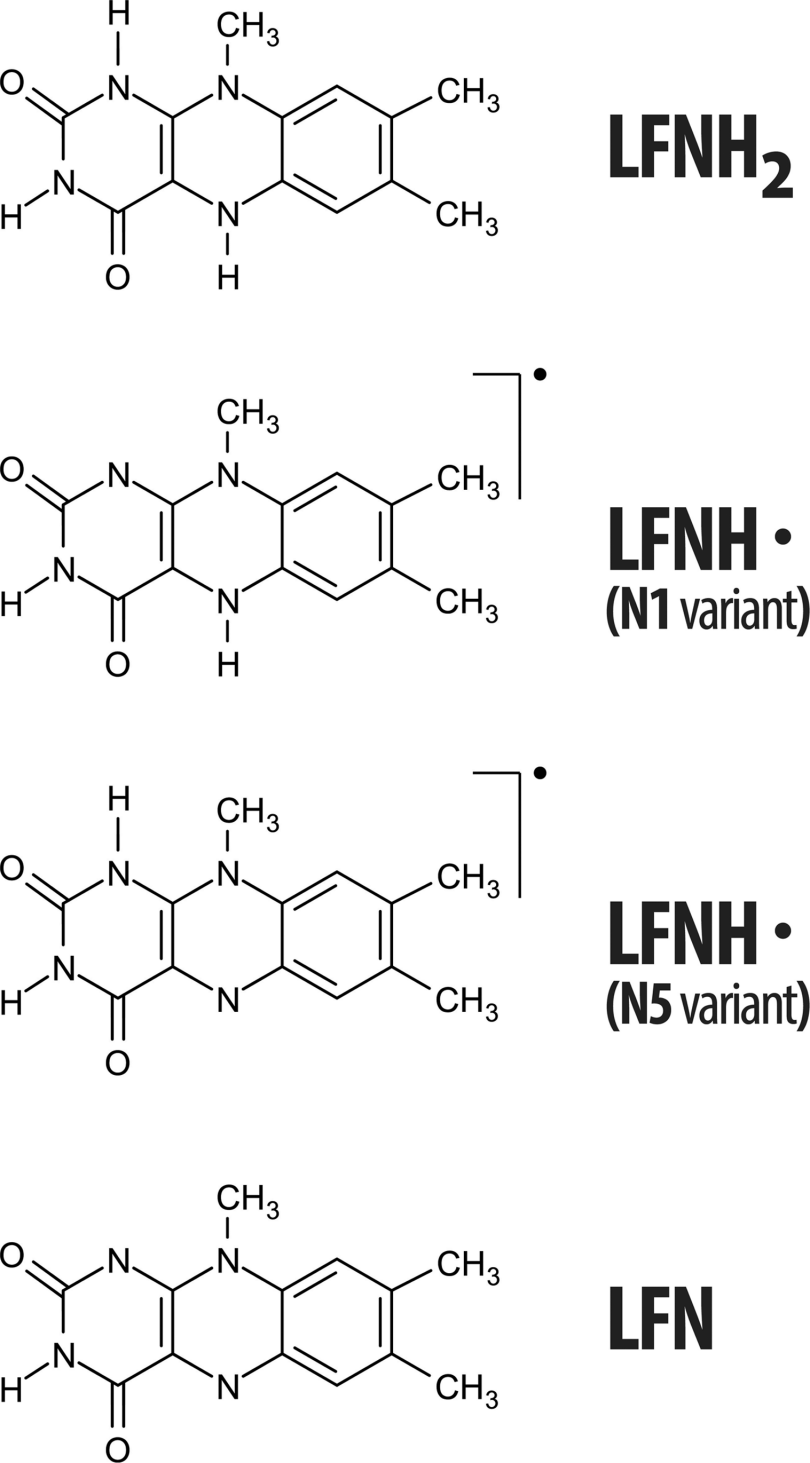
Different forms of LFN taking part in the presently studied oxidation mechanism: LFNH_2_—reduced form; LFNH^•^—semioxidized (radical) form; and LFN—oxidized form. Note that LFNH^•^ appears in two distinct isomers, depending on the site of abstraction of hydrogen atoms; the variant is denoted according to the site from which the first hydrogen has been abstracted (i.e., in the N1 mechanism, the remaining hydrogen is bound to N5 and vice versa in the N5 mechanism).

The order of removal of the two hydrogens from LFNH_2_ was also been considered. As shown in Figure 1, these hydrogens are bound to the N1 and N5 atoms, and the above-listed reaction steps have been modeled in both variants, that is, whenever the LFNH^•^ entity is involved, it is modeled either with the hydrogen atom bound to N1 or N5. Note that the corresponding variants of the mechanism are denoted by the site from which the first hydrogen was abstracted (Figure 4).

An intriguing feature of the presently studied reaction is the fact that molecular oxygen (O_2_) is biradical (spin triplet) in its electronic ground state. As the reaction products are all singlets, the spin state of the system is changed at some point during the course of the reaction. This requires the intersection between the triplet and singlet electronic state be determined, i.e., the challenge is to find a geometry for which the triplet and singlet energy is identical and simultaneously minimize its energy under this condition on both surfaces. Such an optimized crossing point of two surfaces is called minimum energy crossing point (MECP) and the directed search of this point is based on energy gradients with respect to nuclear coordinates on both surfaces. These gradients are combined, forming two orthogonal gradients, one being parallel to the surface crossing hyperline and the other perpendicular to it. At MECP, both gradients are equal to 0.^42,43^ The MECP optimization algorithm is compatible with quantum chemistry approaches capable of computing gradients. In this work, we performed MECP optimizations by using an automated Python script of Rodríguez-Guerra et al.,^44^ which is an improved version of the original code authored by Harvey et al.^43^ By estimating the MECP of two surfaces, one can in principle characterize reaction pathways involving different electronic/spin states. Note however that the change of spin state is a highly complex process requiring quantum dynamics formalisms for a proper treatment. While this is beyond the scope of the present study, we feel that computational characterization of MECPs still provides adequate information about the reaction profiles and kinetics. This aspect will be discussed further in the text.

## 2 Computational Details

All calculations were carried out at the M06-2*X*/6-31+G(d,p) level of theory using the Gaussian 16 program suite.^45^ The M06-2X functional developed by Truhlar and coworkers proved to faithfully describe potential energy surfaces of organic molecules and their reactions.^46−48^ In order to validate the M06-2X functional, we benchmarked it against three other popular functionals often employed in studies of reactivity, namely, B3LYP, BLYP, and PBE. We did so by reoptimizing selected characteristic points on the reaction profile [steps (1) and (5)] in the gas phase. Comparison of the reaction barrier computed by these functionals confirms already reported trends among the functionals,^49^ from which it can be deduced that the M06-2X functional performs quite reliably for the reaction in question. Benchmarking details are presented in Supporting Information, Section S6.

The flavin group was represented by LFN (Figures 3 and 4), which is a common molecular form featuring the isoalloxazine moiety (a structural foundation of flavins). As molecular oxygen (O_2_) exists as a triplet in its electronic ground state, molecular complexes involving O_2_ were in most cases modeled as spin triplets. Complexes involving two radical species (e.g., LFNH^•^ and HOO^•^) were treated both as triplets and singlets, whereas in the case of one radical entity present in the model, a doublet spin state was assumed. For the reaction between LFNH^•^ and O_2_ [step (3)], both the spin quartet and doublet states were assumed. The reduced form of flavin (LFNH_2_) includes two hydrogens plausible for radical abstraction, namely, those bound to N1 and N5 atoms (Figure 3), and the case when the N1 hydrogen is abstracted first is denoted as the N1 variant, whereas the opposite order of abstraction is denoted as the N5 variant. Stationary points on the potential energy surfaces (reactants, products, and transition states) were determined using standard optimization procedures; initial guesses for the transition states were found by running relaxed potential energy surface scans using a selected interatomic distance (e.g., O–O···H) as the control variable. Optimizations to a local minimum or first-order saddle point were verified by a harmonic frequency check. When necessary, convergence of the electronic structure was ensured by the quadratic convergence procedure.^50^ Selected reaction pathways were further characterized by intrinsicreaction coordinate (IRC) calculations^51^ starting from the corresponding transition-state structure. Effects of polar medium were estimated by the SCRF technique using the SMD solvation model^52^ and water as the solvent.

Intersections (MECP’s) between two distinct spin states (i.e., triplet-singlet or quartet-doublet) were determined by the Python script of Rodríguez-Guerra et al.^44^ coupled with Gaussian 16. The script performs a directed MECP search using gradients computed for both spin states for each involved geometry of the system following the algorithm described in refs (42 and 43). As initial guess for MECP optimizations, we used previously computed IRC profiles and/or potential energy scans on both involved surfaces (see for example, Supporting Information, Figure S19) on which we visually estimated the intersection and extracted the corresponding molecular geometry. Effects of polar medium were estimated by the SCRF technique using the SMD solvation model^52^ and water as the solvent.

## 3 Results and Discussion

In this section, the aforementioned five steps (see Introduction) comprising the radical mechanism will be analyzed and discussed. The reader is referred to the Supporting Information for a more detailed presentation of the steps. These steps can be combined in several ways, but usually two steps are sufficient to acquire the final product, that is, lumiflavin in an oxidized form (LFN). We use the following convention for the reference energy: for each step, the zero energy value is defined by infinitely separated (isolated) molecules on the reactant side. Consequently, when investigating a selected step as part of a sequence of steps, the energy of separated products of the precedent step has to be added to the energy profile of that step in order to properly estimate its energetics within the sequence.

### 3.1 Step (1): LFNH_2_ + O_2_ → LFNH^•^ + HOO^•^

In a triplet state, LFNH_2_ and O_2_ can form a weakly bound complex in a variety of conformations that barely differ in energy. Taking the optimized separated molecules in their ground electronic state as a reference, the complex is stabilized by approximately 1.5 kcal/mol both in the gas phase and in solution (Table 1). Abstraction of the N1 or N5 hydrogen leads to the transition state with quite different barriers for the N1 and N5 variants of 15.2 and 21.3 kcal/mol, respectively. This reaction step is slightly endergonic, the product complex LFNH^•^···HOO^•^ being at 2.0 and 9.0 kcal/mol for the N1 and N5 variant, respectively. Dissociation of the product complex to separated LFNH^•^ and HOO^•^ radicals requires 17.2 (Supporting Information, Figure S1) and 26.1 kcal/mol (Supporting Information, Figure S2) relative to the separated reactants for N1 and N5 abstraction, respectively. Implicit solvent reaction field noticeably lowers the barrier and stabilizes the products of this step for both abstraction sites. This is most probably due to the changes in charge distribution along the course of the reaction during which the polarity of the system gradually increases, as reflected in the dipole moment increasing from ∼6 D in reactants to ∼11 D in products. Application of the singlet spin state drastically increases the energy of all involved entities—typically by over 30 kcal/mol—rendering this reaction step highly disfavored for spin-paired electronic configuration (see the Supporting Information for details).

**Table 1:** Energies of Characteristic Structures Involved in the LFNH_2_ + O_2_ → LFNH^•^ + HOO^•^ Reaction [Step (1)] Computed for Both Abstraction Variants (N1 and N5), as Well as for an Isolated (Gas) and Implicit Solvation (SCRF) Model^a^

variant	reactant complex		transition state		product complex		dissociated products	
	gas	SCRF	gas	SCRF	gas	SCRF	gas	SCRF
N1	–1.5	–1.6	15.2	7.4	2.0	–1.1	17.2	7.6
N5	–1.7	–1.3	21.3	6.9	9.0	3.7	26.1	13.4

a All values are given in kcal/mol.

Summarizing the above characteristics, the N1 abstraction variant is preferred over N5, and solvation considerably lowers the production cost of LFNH^•^ and HOO^•^ radicals. Characteristic energies of both variants are listed in Table 1.

Importantly, step (1) represents the source of radical species which may undergo the next reaction steps in various combinations, and the two rightmost columns of Table 1 (dissociated products) list the required energy input depending on the abstraction site and medium. These values should be added to energy profiles of subsequent steps in order to assess the overall energetics properly.

### 3.2 Step (2): LFNH_2_ + HOO^•^ → LFNH^•^ + H_2_O_2_

From the kinetics standpoint, this step is irrelevant because (i) it requires step (1) be done in advance to provide the HOO^•^ radicals required at the reactant side and (ii) the main product of this step—the LFNH^•^ radical—is the same as in step (1). This means that whatever the barrier of this step, characteristics of step (1) will prevail. Nevertheless, some details of this step merit consideration and are presented in the Supporting Information.

### 3.3 Step (3): LFNH^•^ + O_2_ → LFN + HOO^•^

This step has potential relevance for overall reaction kinetics because it yields the final product, that is, LFN in oxidized form. Furthermore, both reacting entities are spin-unpaired on their own (LFNH^•^ is a spin doublet, whereas O_2_ is a triplet), and on interaction, their spins can combine to form a doublet or they can barely interact so that the system is in a quartet spin state. The products of this step include the spin-paired LFN molecule and the HOO^•^ radical which exists as a doublet in its ground state; therefore, a potential quartet spin state must convert to doublet during this step. In this respect, we found substantial difference between the reaction in the gas phase and in an aqueous environment. Namely, in the gas phase, the LFNH^•^···O_2_ reacting complex has virtually identical energy (within ∼0.03 kcal/mol precision) in both quartet and doublet spin state. Gas-phase MECP calculations fully support this, confirming the reactant complex as converged MECP of the quartet and doublet surface. All of the subsequent stages of this step preferably exist in the doublet state. In contrast to this, in the solution, transition between the spin states apparently occurs later during the course of reaction; structures corresponding to the crossing between the, respective, surfaces are reasonably close to the transition state structure in the gas phase.

Table 2 lists energies of the characteristic species involved in the profiles. Note that while the entire profile in the gas phase corresponds to the doublet state, the one in the implicit solvent represents a minimum energy path connecting reactants in the quartet spin state and products in the doublet state, with the quartet-doublet MECP being the highest point of that profile, representing an estimate of the barrier. For both types of environment, the N5 variant is kinetically and thermodynamically preferable by a large margin, which is in agreement with what has been found for steps (1) and (2), in which the hydrogen bound to N1 requires less energy input to be abstracted. One should mind that when LFNH^•^ is the reacting species with one hydrogen already abstracted and one yet to be removed, it is the hydrogen at N5 to be removed in the N1 variant of the mechanism, and vice versa.

**Table 2:** Energies of Characteristic Structures Involved in the LFNH^•^ + O_2_ → LFN + HOO^•^ Reaction [Step (3)] Computed for Both H-Abstraction Variants (N1 and N5), as Well as for an Isolated (Gas) and Implicit Solvation (SCRF) Model: (i) Reactant Complex (Gas Phase: Doublet State; SCRF: Quartet State); (ii) Gas Phase: Transition State; (ii) SCRF: MECP between the Quartet and Doublet Potential Energy Surface; and (iii) the Product Complex in the Doublet State^a^

gas phase						
variant	energy relative to separated reactants of step (3) (LFNH^•^ + O_2_)			energy relative to separated reactants of step (1) (LFNH_2_ and O_2_)		
	(i) reactant complex	(ii) transition state	(iii) product complex	(iv) cost to form LFN^•^ radicals (step (1)/Table 1)	(v) total barrier	(vi) total energy
N1	–2.1	26.3	–4.6	17.2	43.5	12.6
N5	–1.5	13.1	–13.3	26.1	39.2	12.8

SCRF						
variant	energy relative to separated reactants of step (3) (LFNH^•^ + O_2_)			energy relative to separated reactants of step (1) (LFNH_2_ and O_2_)		
	(i) reactant complex	(ii) quartet-doublet MECP	(iii) product complex	(iv) cost to form LFN^•^ radicals (step (1)/Table 1)	(v) total barrier	(vi) total energy
N1	–2.5	10.8	–3.7	7.6	18.3	3.9
N5	–3.3	1.3	–11.3	13.4	14.7	2.1

a For the gas-phase model, the entire reaction pathway is in the double spin state, whereas for the SCRF model, the minimum energy path of the reaction includes the spin change from quartet to doublet at its highest point. Entry (iv) includes the energy cost for the formation of LFNH^•^ radicals produced in step (1), whereas (v) is the total barrier estimate of successive steps (1) and (3), which is the sum of terms in (ii) and (iv). Entry (vi) is the reaction energy of successive steps (1) and (3), obtained by summing of (iii) and (iv). All values are given in kcal/mol.

A sequence of steps (1) and (3) facilitates the formation of the final product out of the initial reacting species. Addition of the cost to form isolated LFNH^•^ radicals yields effective barriers of 43.5 and 39.2 kcal/mol (for the N1 and N5 variants, respectively) for the gas phase, and 18.3 or 14.7 kcal/mol in the solution for the same sites. This suggests that the kinetics of such mechanism is rather slow in the gas phase but may be feasible on the same time scale as observed in the MAO-B enzyme, particularly the N5 variant (note that in the active site of the MAO-B enzyme, the effective barrier for flavin oxidation is deduced to be in the range of ∼15 kcal/mol). Nevertheless, particularly due to the large mismatch in the gas phase barrier that is at best at ∼40 kcal/mol, one should inspect whether other, kinetically more favorable mechanisms are in operation.

### 3.4 Step (4): LFNH^•^ + LFNH^•^ → LFN + LFNH_2_

This is a termination step since radicals are consumed, forming spin-paired products. Conceptually, two LFNH^•^ radicals (semioxidized form of LFN) exchange a hydrogen atom in the form of H^•^ radicals, yielding one reduced (LFNH_2_) and one oxidized (LFN) lumiflavin molecule. The computed profiles based on MECP optimizations (mind that this reaction includes the change of spin state from triplet to singlet) suggest that the reaction may be kinetically feasible with effective barriers reasonably low (20.9 and 6.7 kcal/mol for the gas phase and implicit solvent, respectively), but at the same time, a reaction involving two flavin entities can hardly occur in the protein matrix. Therefore, while a mechanism involving step (4) cannot be regarded as reasonable in the context of enzyme function or regeneration, it includes details worth consideration. Step (4) is presented and discussed in the Supporting Information.

### 3.5 Step (5): LFNH^•^ + HOO^•^ → LFN + H_2_O_2_

This step is a complete termination of the reaction, yielding both final products from intermediates released in step (1). As such, together with precedent step (1), it represents the most straightforward course of the reaction. In addition, this step appears to be favorable from both kinetic as well as thermodynamic standpoint. Like in steps (3) and (4), there is a change of spin state (from triplet to singlet) during the reaction, and the intersection between the, respective, surfaces represents the highest point on the minimum energy path. Similar to previous steps, that crossing point is an estimate of the effective barrier of step (5) but not necessarily of the total reaction composed of successive steps (1) and (5). While in the triplet state the reaction proceeds over a regular transition state to energetically elevated products, the process is of a barrierless-downhill type in the singlet state, starting at energetically disfavored reactants but relaxing rapidly toward products. Energies of characteristic entities along the minimum energy path are listed in Table 3.

**Table 3:** Energies of Characteristic Structures Involved in the LFNH^•^ + HOO^•^ → LFN + H_2_O_2_ Reaction [Step (5)] Computed for Both H-Abstraction Variants (N1 and N5), as Well as for an Isolated (Gas) and Implicit Solvation (SCRF) Model: (i) Reactant Complex in the Triplet State; (ii) the MECP between the Triplet and Singlet Potential Energy Surface; and (iii) the Product Complex in the Singlet State^a^

variant	energy relative to separated reactants of step (5) (LFNH^•^ + HOO^•^)						energy relative to separated reactants of step (1) (LFNH_2_ and O_2_)					
	(i) reactant complex (triplet)		(ii) triplet-singlet MECP		(iii) product complex (singlet)		(iv) cost to form radicals (step (1)/Table 1)		(v) MECP energy (crossing + step 1)		(vi) total energy (product complex + step 1)	
	gas	SCRF	gas	SCRF	gas	SCRF	gas	SCRF	gas	SCRF	gas	SCRF
N1	–18.7	–9.0	–3.5	–5.7	–33.5	–32.6	17.2	7.6	13.7	1.9	–16.3	–25.0
N5	–19.6	–9.5	–3.4	–5.0	–43.7	–37.4	26.1	13.4	22.7	8.4	–17.6	–24.0

a These stages are part of the minimum energy path of the reaction accompanied by the spin change from triplet to singlet. Entry (iv) includes the energy cost for the formation of radicals produced in step (1), whereas (v) is the total barrier estimate of successive steps (1) and (5), which is the sum of terms in (ii) and (iv). Entry (vi) is the reaction energy of successive steps (1) and (5), obtained by summing (iii) and (iv). All values are given in kcal/mol.

As the triplet-singlet crossing is located at fairly low energies, the effective barriers of step (5) are considerably lower than those in step (3). Therefore, unlike step (3), the MECPs of step (5) do not necessarily represent the effective barrier of a net reaction consisting of successive steps (1) and (5). In order to devise the barrier of the net reaction and pinpoint the rate-limiting factor, Table 4 includes comparison of triplet-singlet MECP energies of step (5) with barriers of step (1), both expressed relative to the same reference state, i.e., energy of isolated reactants LFNH_2_ and O_2_.

**Table 4:** Comparison of the Computed Transition State (TS) of Step (1) and Triplet-Singlet MECP of Step (5) for Both Variants of Hydrogen Abstraction Order and for Both Media^a^

variant	TS of step (1)	MECP of step (5)
N1, gas	15.2	13.7
N5, gas	21.3	22.7
N1, SCRF	7.4	1.9
N5, SCRF	6.9	8.4

a The value indicating the rate-limiting step of the reaction consisting of successive steps (1) and (5) is displayed in bold underlined formatting for each variant of the reaction.

In both media, the N1 variant features the barrier of step (1) as the rate-limiting factor, whereas in the N5 variant, the triplet-singlet MECP of step (5) represents the highest point of the reaction profile. However, with the exception of the N1 variant in solution, the energy difference between the TS and MECP is very low, in the range between 1.0 and 1.5 kcal/mol, leaving the question of the effective rate-limiting step open—we feel that this difference does not exceed the inaccuracy inherent to the model.

As the triplet-singlet crossing is located at fairly low energies, the effective barriers are considerably lower than that in step (3). Taking into account the cost of formation of radicals in step (1), the gas-phase barrier amounts to 15.2 and 22.7 kcal/mol for the N1 and N5 variants, respectively, rendering the N1 variant clearly preferential. In contrast to that, in a polar aqueous environment, the barrier is substantially lower and much less distinct between the two variants, namely, 7.4 and 8.4 kcal/mol, respectively. The reaction consisting of successive steps (1) and (5) is by far the most exergonic among all considered combination of steps (around −17 kcal/mol in the gas phase and around −25 kcal/mol in solution).

**Figure 5:**
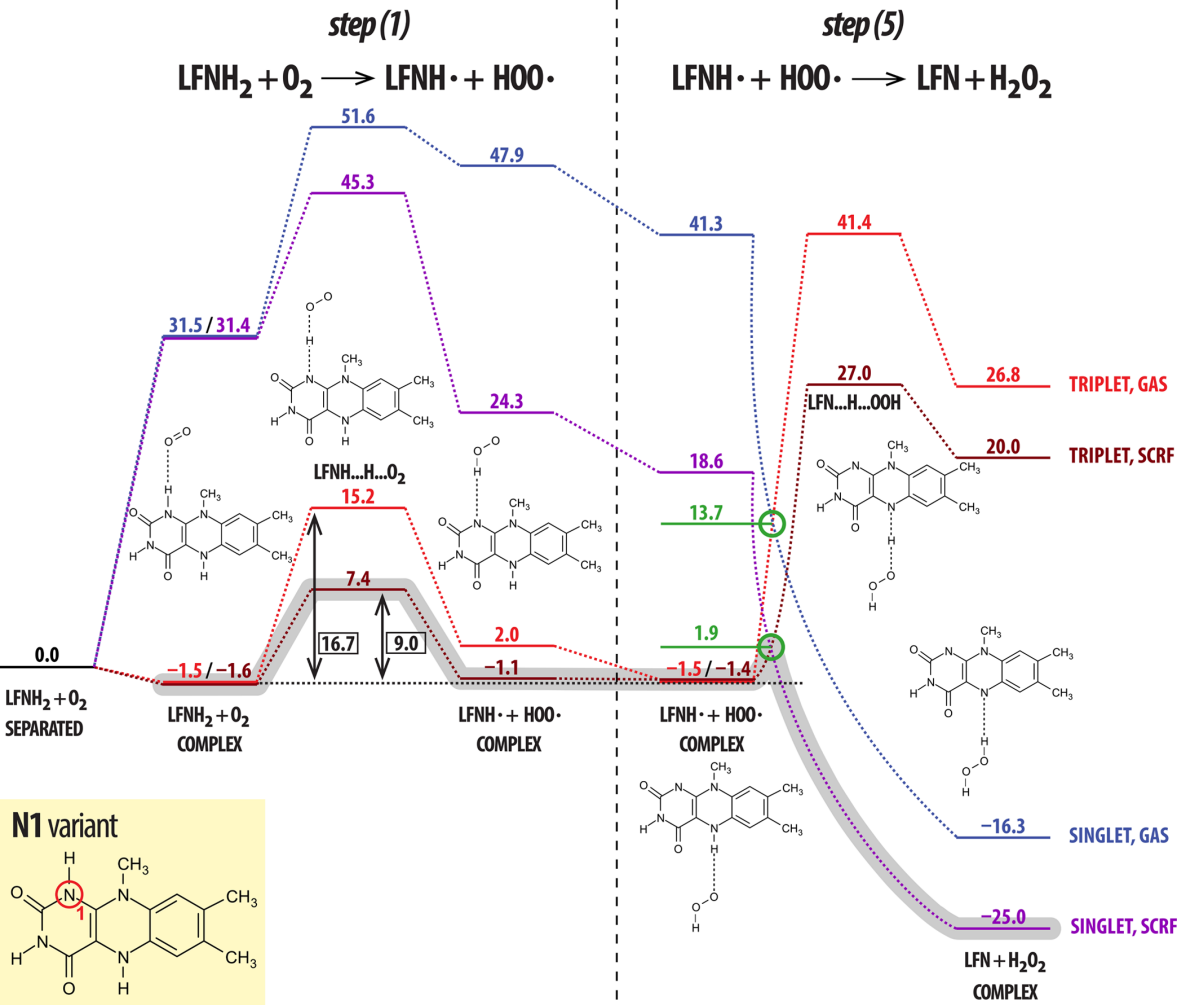
Schematic representation of oxidation of flavin proceeding by a radical mechanism (N1 variant) consisting of steps (1) and (5). Color code: red—triplet spin state in the gas phase; brown—triplet spin state, implicit solvation model (SCRF; water as the solvent); blue—singlet spin state in the gas phase; and purple—singlet spin state, implicit solvation model. The MECP between the triplet and singlet states is shown in green for both phases. All displayed values are energies (in kcal/mol) of the corresponding species given relative to the isolated reactants in their ground state, except for the framed black values which correspond to the effective barrier in the gas phase and in the solvent reaction field (given relative to the LFNH_2_···O_2_ reactant complex). The minimum energy path combining both spin states is outlined in light gray for the solution model.

For the sake of comparison and evaluation of the three possible mechanisms, their kinetic and thermodynamic parameters are summarized in Table 5. The preferred mechanism in both media is schematically displayed in Figure 5.

**Table 5:** Barriers and Energies of LFN Oxidation Proceeding by a Radical Mechanism in Various Combinations of Steps as Presented above for Both the Isolated (Gas) and the Implicit Solvation (SCRF) Model^a^

reaction steps	preferred variant	reaction barrier [kcal/mol]		reaction energy [kcal/mol]	
		gas	SCRF	gas	SCRF
step (1) + step (3)	N5	40.7	16.3	12.8	2.1
step (1) + step (5)	N1	16.7	9.0	–16.3	–25.0

a For each of the three combinations, the most favorable among the variants (e.g., N1 vs N5) is listed. While the reaction energies are given relative to the energy of separated initial reactants (LFNH_2_ and O_2_), the barrier is given relative to the energy of the initial reacting complex (LFNH_2_···O_2_) in the corresponding state (gas phase or SCRF).

It should be noted that while the listed reaction energies are directly reproduced from Tables 2, 3, and 4 for the, respective, mechanisms, the barriers have been corrected for the energy of the LFNH_2_···O_2_ reactant complex which has a slightly lower energy than separated reactants (by approximately 1.5 kcal/mol, depending on the hydrogen abstraction site and environment; see Table 1). This means that the actual barriers are higher by that amount so as to span the range between the reactant complex and the highest point on the minimum energy profile.

The mechanism involving consecutive steps (1) and (5) is shown as the most favorable both kinetically (lowest barrier in both media) and thermodynamically (most exergonic). While the barrier estimate in the gas phase is in very good qualitative agreement with experimental kinetic assessment of MAO enzymes, the barrier evaluated in the aqueous solution is noticeably lower. Because the polar enzymatic environment is very complex, it may affect the barrier in a variety of different ways in different enzymes; therefore, any comparison with the rather simplified implicit solvation model is of limited value. Nevertheless, despite the radical mechanism, the involved entities are apparently of sufficiently high polarity to be prone to effects of the polar/electrostatic interactions exerted by the solvent or by an enzyme.

## 4 Conclusions

We investigated oxidation of flavin by molecular oxygen, which is a highly relevant reaction involved in many life processes because a number of enzymes use flavin as a cofactor, mainly in oxidoreductive reactions. In such enzymes, flavin oxidation is required to restore the catalytic capabilities of an enzyme, thus completing the catalytic cycle. Our work is based on quantum chemistry protocols and is focused solely on the radical mechanism. We decomposed the net reaction to several steps involving spin-unpaired entities and characterized these steps by finding stationary points on the, respective, potential energy surfaces and evaluating the pathways between them. As the change of spin state during the reaction course is by definition mandatory already for the net reaction (because reactants include molecular oxygen in a triplet state, whereas all products are spin-paired), we attempted to characterize the intersections between the, respective, surfaces. Importantly, we found that the MECP between two different spin states likely represents the effective barrier not only for the reaction step under investigation but may also be relevant for the net reaction. By comparing three different reaction pathways including five possible steps, we found that the thermodynamically and kinetically preferred mechanism consists of two successive abstractions of hydrogen from the flavin rings, the first one being cast by molecular oxygen, yielding semioxidized flavin and hydroperoxyl radicals; in the next step, these radicals rearrange, facilitating abstraction of the second hydrogen from the flavin ring by hydroperoxyl radicals (Figure 5). The rate-limiting factor of this process in the gas phase is likely associated with the barrier of abstraction of the first hydrogen (preferably N1), but due to small energy differences, the change of the spin state from triplet to singlet during the abstraction of second hydrogen cannot be excluded as a rate-limiting factor. For both models, the reaction is exergonic, the products being at about 16 and 25 kcal/mol below isolated reactants, respectively. Other investigated mechanisms feature considerably higher barriers and lower exergonicity (see Table 5). All mechanisms exhibit dependence on the polar environment, in which polar solvent decreases the barrier and increases exergonicity—possibly due to the fact that polarity of the system gradually increases during the reaction, particularly during the initialization step. The order of abstraction of the two hydrogens from the flavin molecule has also been considered, revealing the N1 site to be preferred over N5; however, this effect is partially (and in some cases, entirely) canceled out on abstraction of the second hydrogen.

Both in the gas phase and in aqueous solution, the estimated barriers of the preferred mechanism (∼17 and ∼9 kcal/mol, respectively) can be regarded as reasonable. Namely, kinetic studies of related MAO enzymes suggest that the flavin regeneration barrier is of similar magnitude as that of the reactive step (MAO-B), or lower (MAO-A), implying that the flavin oxidation barrier is in the range of ∼15 kcal/mol. The higher gas-phase barrier is understandable in which the polar environment reportedly lowers the barrier to some extent,^53^ suggesting lower barriers also in enzyme active sites. At the same time, the gas-phase barrier estimate is in the range suggested by the experiment, while the barrier computed in the polar solvent (∼9 kcal/mol) is somewhat low but not in utter disagreement with experimental evidence either. This supports the view that a radical mechanism may be in operation in oxidative regeneration of flavin, but one should mind that reactivity in an enzymatic environment includes very complex effects not undertaken in the present study.

Several caveats possibly impacting the above assessment should be mentioned. First, although the presently investigated reaction mechanisms appear to be conceptually simple, many parts have been exceedingly challenging for a proper treatment even with routine protocols. Particularly in the case of SCRF calculations, optimizations of transition states (and sometimes other stationary points) often failed to achieve convergence. For that sake, we chose a single-point calculation imposed on the gas-phase stationary point rather than SCRF treatment with included full optimization. We sometimes observed similar difficulties with higher spin states. In certain cases, even single-point energy evaluations were demanding, but we resolved this issue by applying a robust, reliable but computationally expensive quadratic convergence SCF procedure.^50^ This suggests that the potential energy surfaces pertinent to the reaction are very complex. In fact, the complexity of the surfaces has been among the reasons for focusing our investigation solely on a radical mechanism, thereby leaving aside alternative polar mechanism(s). One such possibility is a mechanism involving a molecular complex between LFN and O_2_ (with a variant of oxygen covalently bound to *C*4a and *C*10a of LFN) in which both N1 and N5 hydrogens are transferred in a concerted manner to oxygen in the form of proton/hydride, possibly assisted by bridging water molecules. Our preliminary evaluation suggests that such mechanisms are worth considering, but at the same time, similar difficulties related to the complexity of the reaction surface are expected. In this regard, improvements and additional studies can be foreseen but are beyond the scope of this work. Here, it should be noted that we also omitted from our treatment the caged radical pair transformation to the final product via the flavin-*C*4a-hydroperoxide intermediate [reactions 3 and (4) in Figure 2]. Although this route has been suggested to be involved in many cases of enzyme-mediated flavin oxidation, the fact that it involves a C–O bond cleavage accompanied by hydrogen transfer from N5 to the departing hydroperoxyl group renders it exceedingly challenging for the present treatment. Also, the mechanism depicted in Figure 2 features a N1-deprotonated flavin molecule already at the onset, essentially adding the very demanding protolysis (p*K*_a_) issue to the treatment. From our initial assessment of several variants of the mechanism, including those displayed in Figure 2, the barrier related to the change of spin from triplet to singlet in the involved entities appears to be significantly higher than the one presently found for successive steps (1) and (5), but this remains to be verified by future studies.

Another obvious limitation is the omission of an explicit enzymatic environment that can crucially control the kinetics and thermodynamics of flavin oxidation. Again, in conjunction with the complexity of the potential energy surface, inclusion of neighboring residues in DFT calculations would likely improve reliability^54^ but at the same time render the treatment exceedingly difficult (not to mention persisting limitations of an expanded cluster model, in which it can barely account for the complete electrostatics). Nevertheless, the findings of the present study provide guidelines that will be important for future research of the reaction mechanism. This also applies to the possibility of embedding the above-elucidated mechanism and its rate-limiting step [step (5)] into a fully scaled enzymatic environment by using the EVB^55^ or another QM/MM technique. In this regard, evaluation of the matrix coupling element required for characterization of transition kinetics between the Born–Oppenheimer surfaces would be particularly demanding.

Another limiting factor is the fact that DFT methods are probably slightly less accurate for excited electronic states. The choice of DFT for the present study derives from the fact that DFT has been massively and successfully used in the modeling of a wide variety of related chemical reactions,^41^ including those in biomolecular systems and also involving flavin structure and chemistry.^26^ In addition, our experience with DFT modeling of (enzymatic) reactions involving flavin is exclusively positive.^4,53,56^ We are aware that the present investigations of higher spin states should be taken with caution, but at the same time, we stress that full engagement of superior post-Hartree–Fock electronic structure techniques to the presently studied system (35 atoms and 152 electrons) exceeds our available resources by quite a large margin.

As the presently studied reaction includes transition(s) between different electronic/spin states, it should be noted that the transition mechanism is a highly complex dynamic phenomenon requiring time-dependent quantum treatment for a full assessment of the factors governing the kinetics. Again, such a treatment is out of the scope of the present work. Nevertheless, we stress that a detailed investigation of parts of the potential energy surfaces related to these transitions is a mandatory prerequisite for deeper insights into the transition dynamics. As such, the present study represents a valuable resource for future efforts in this direction.

## Supporting Information Available

Detailed description and graphical representation of mechanism and geometric features of all reaction steps presented in the article; benchmark calculations and justification of the presently used M06-2X functional; and xyz coordinates of all relevant entities (stationary points and MECPs) involved in all reaction steps (PDF)
Step1-N1-R (XYZ)
Step1-N1-TS (XYZ)
Step1-N1-P (XYZ)
Step1-N5-R (XYZ)
Step1-N5-TS (XYZ)
Step1-N5-P (XYZ)
Step2-N1-P (XYZ)
Step2-N5-R (XYZ)
Step2-N5-TS (XYZ)
Step2-N5-P (XYZ)
Step3-N1-R (XYZ)
Step3-N1-TS-gas (XYZ)
Step3-N1-MECP-scrf (XYZ)
Step3-N1-P (XYZ)
Step3-N5-R (XYZ)
Step3-N5-TS-gas (XYZ)
Step3-N5-MECP-scrf (XYZ)
Step3-N5-P (XYZ)
Step4-v51-R (XYZ)
Step4-v51-MECP-gas (XYZ)
Step4-v51-MECP-scrf (XYZ)
Step4-v51-P (XYZ)
Step5-N1-R (XYZ)
Step5-N1-MECP-gas (XYZ)
Step5-N1-MECP-scrf (XYZ)
Step5-N1-P (XYZ)
Step5-N5-R (XYZ)
Step5-N5-MECP-gas (XYZ)
Step5-N5-MECP-scrf (XYZ)
Step5-N5-P (XYZ)

## Supplementary Material

ao4c00307_si_001.pdf

ao4c00307_si_002.xyz

ao4c00307_si_003.xyz

ao4c00307_si_004.xyz

ao4c00307_si_005.xyz

ao4c00307_si_006.xyz

ao4c00307_si_007.xyz

ao4c00307_si_008.xyz

ao4c00307_si_009.xyz

ao4c00307_si_010.xyz

ao4c00307_si_011.xyz

ao4c00307_si_012.xyz

ao4c00307_si_013.xyz

ao4c00307_si_014.xyz

ao4c00307_si_015.xyz

ao4c00307_si_016.xyz

ao4c00307_si_017.xyz

ao4c00307_si_018.xyz

ao4c00307_si_019.xyz

ao4c00307_si_020.xyz

ao4c00307_si_021.xyz

ao4c00307_si_022.xyz

ao4c00307_si_023.xyz

ao4c00307_si_024.xyz

ao4c00307_si_025.xyz

ao4c00307_si_026.xyz

ao4c00307_si_027.xyz

ao4c00307_si_028.xyz

ao4c00307_si_029.xyz

ao4c00307_si_030.xyz

ao4c00307_si_031.xyz
