# Supplementary material for: Oxidation of Flavin by Molecular Oxygen: Computational Insights into a Possible Radical Mechanism
Source: ACS Omega. 2024 May 22;9(22):23431–41. doi: 10.1021/acsomega.4c00307 (PMC11154890; doi:10.1021/acsomega.4c00307)
Supplement: Supplementary file 1 — ao4c00307_si_001.pdf [file ao4c00307_si_001.pdf]

Oxidation of flavin by molecular oxygen: computational insight into a possible radical mechanism

*Jernej Stare**

Theory department at the National Institute of Chemistry, Ljubljana, Slovenia

**Corresponding author, e-mail : jernej.stare@ki.si*

SUPPLEMENTARY INFORMATION

S1. Details on step (1): $LFNH_2 + O_2 \rightarrow LFNH\bullet + HOO\bullet$

Energetics of this step is schematically presented in Fig. S1 and S2 for the *N*1 and *N*5 abstraction variant, respectively, whereas geometric features of the corresponding stationary points are displayed in Fig. S3 and S4. In the gas-phase the reactant complex is stabilized (relative to separated molecules) by 1.5 kcal/mol (Fig. S1) when O_2 interacts with the *N*1 hydrogen and by 1.7 kcal/mol when it is in proximity of the *N*5 hydrogen (Fig. S2). In both variants the N—H…O motif is almost linear with the O…N distance of about 3.3 Å (Fig. S3 and S4). In agreement with the low interaction energy, these distances correspond to a very weak hydrogen bond. In the transition state the O…N distance is strongly reduced, namely to roughly 2.4 and 2.5 Å for *N*1 and *N*5, respectively (Figs. S3-4). The N—H bond is significantly elongated towards the midpoint, but the hydrogen atom still resides closer to N than to O. For both abstraction sites, the elongated N—H bond deviates from the plane formed by flavin rings. Transition states of *N*1 and *N*5 abstraction are by 15.2 (Fig. S1) and 21.3 kcal/mol (Fig. S2), respectively, above the reference state (separated molecules), exhibiting sizeable difference between abstraction sites; this is in part reflected in slightly different geometry of the N—H…O moiety (Figs. S3-4). Moving to the product well of this step, the stable LFNH…HOO complex is higher in energy than the complex of reactants – its energy relative to isolated molecules is 2.0 (Fig. S1) and 9.0 kcal/mol (Fig. S2) for N1 and N5, respectively, further enlarging the difference between *N*1 and *N*5 abstraction. However, the interaction between the semi-oxidized flavin and hydroperoxyl radical appears to be stronger: the O…N distance of about 2.7 and 2.8 Å for *N*1 and *N*5 site is significantly shorter than in reactants (Figs. S3-4). Consequently, disintegration of the LFNH…HOO complex is energetically more costly than for the reactant ($LFNH_2…O_2$) complex, and the energy of the separated LFNH• and HOO• radicals is by 17.2 (Fig. S1) and 26.1 kcal/mol (Fig. S2) above the separated reactants for *N*1 and *N*5 abstraction, respectively. Note that the difference of 8.9 kcal/mol between the two abstraction sites is solely due to the difference in stability between the two isomers of LFNH•. During the reaction the O…O distance in the oxygen molecule gradually increases from 1.2 to 1.3 Å on conversion to the hydroperoxyl radical, which is an evident and expected effect.

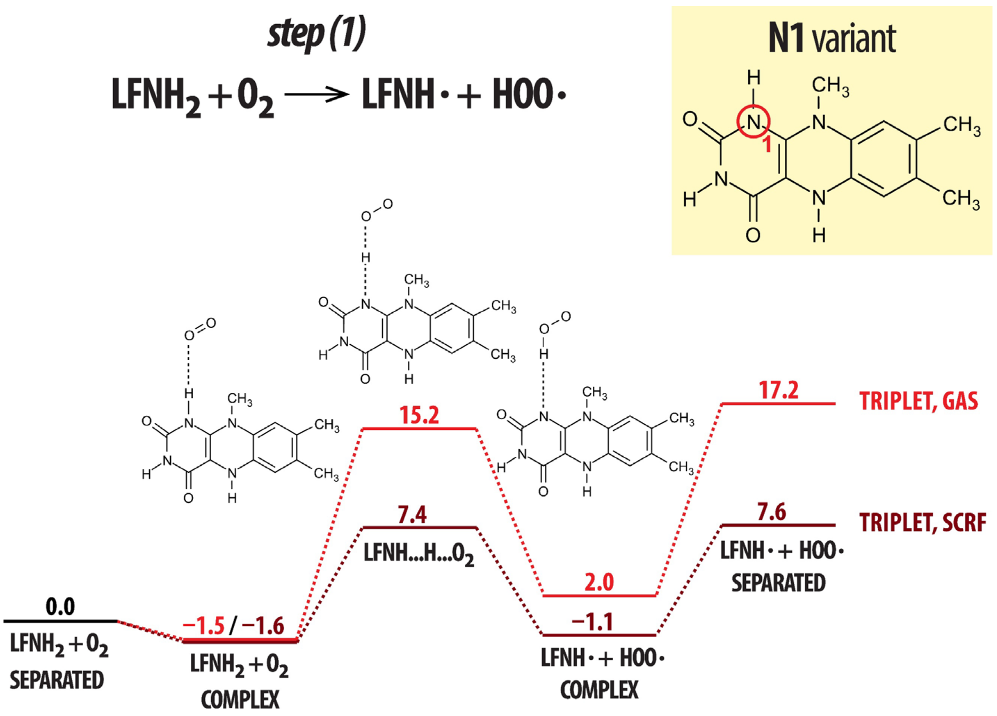

Figure S1. Schematic representation of initialization (step 1) of oxidation of flavin (*N*1 variant). Color code: red – triplet spin state in the gas phase; brown – triplet spin state, implicit solvation model (SCRF; water as solvent). All energies are given in kcal/mol relative to the separated reactant molecules in the respective phase (gas or SCRF).

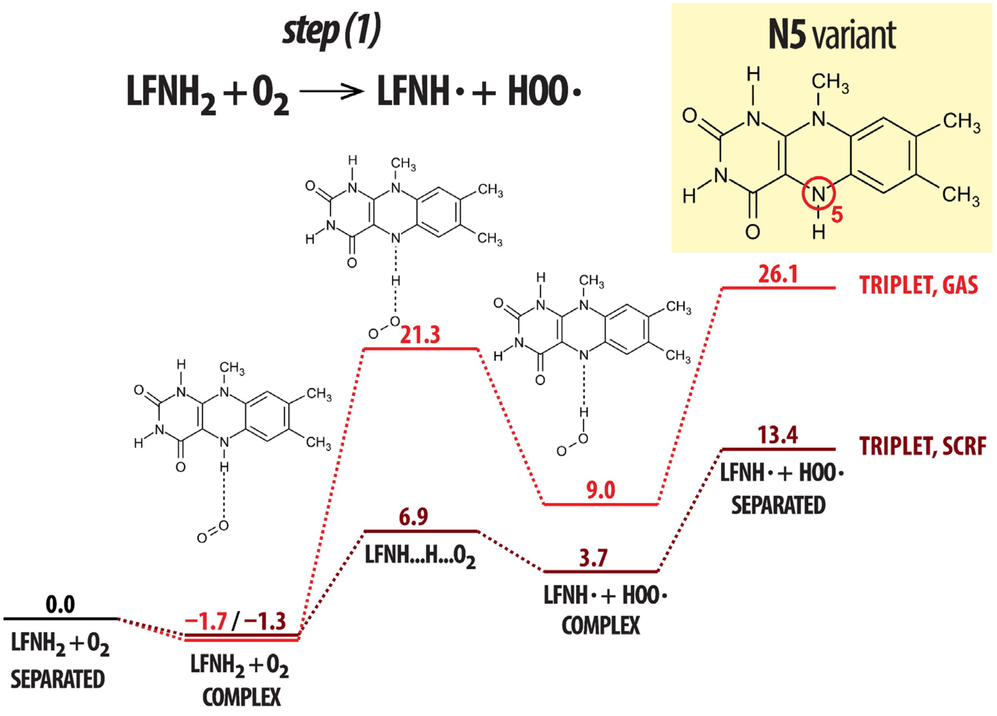

Figure S2. Schematic representation of initialization (step 1) of oxidation of flavin (*N*5 variant). Color code and energy definition are the same as in Fig. S2.

As mentioned in the main text, polarity (dipole moment) of the system increases during reaction, therefore polar environment stabilizes the transition state and the product complex to a larger extent relative to reactants, hence the barrier is significantly reduced (Figs. S1-2).

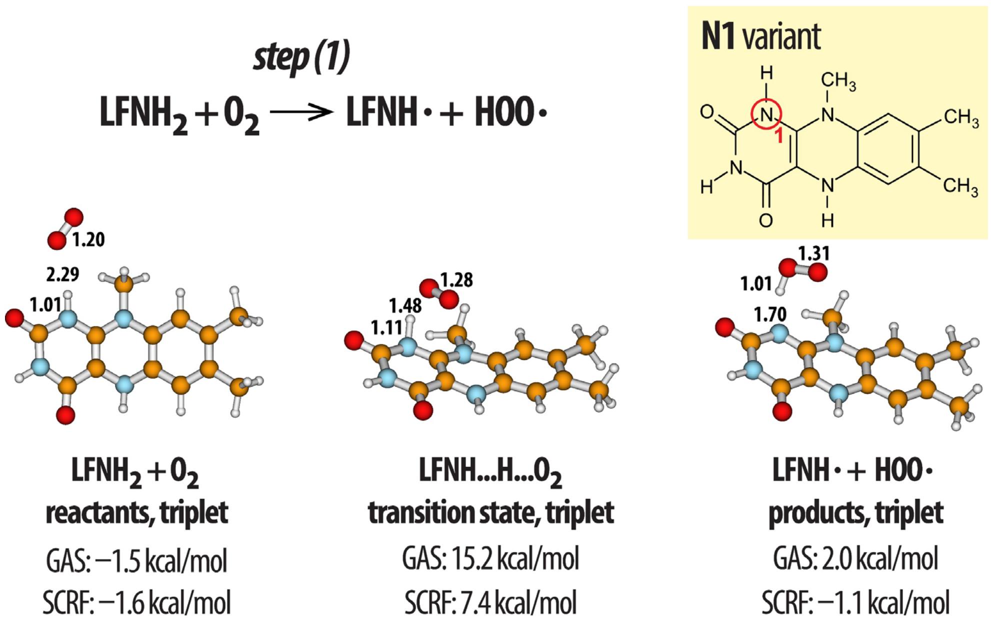

Figure S3. Characteristic structures involved in initialization (step 1) of oxidation of flavin (*N*1 variant) together with N…H, H…O and O…O distances (in Å). Energies of entities (relative to separated reacting molecules) for both the isolated and SCRF model are also displayed.

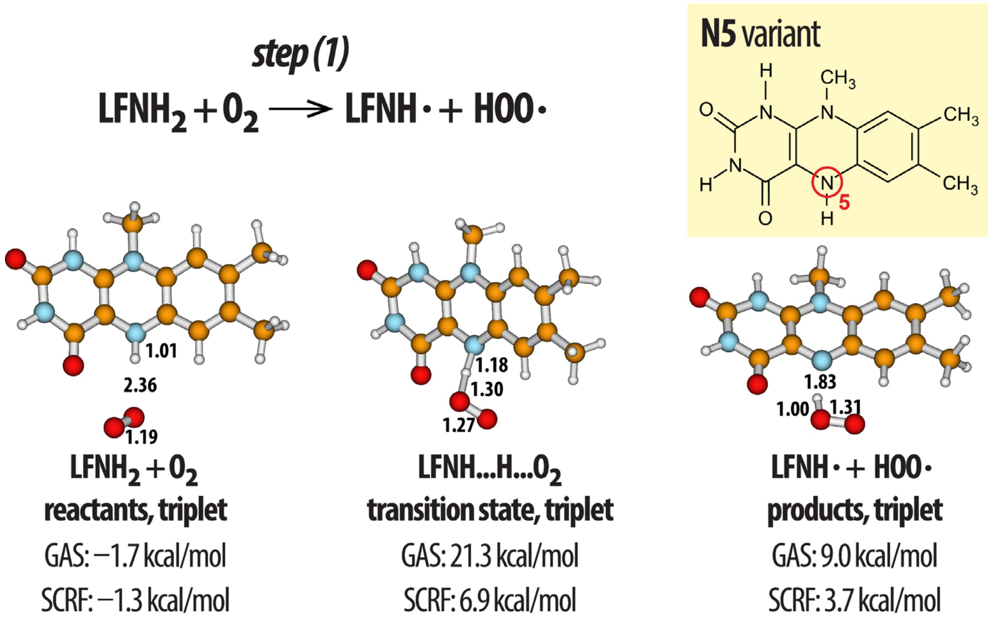

Figure S4. Characteristic structures involved in initialization (step 1) of oxidation of flavin (*N*5 variant) together with N…H, H…O and O…O distances (in Å). Energies of entities (relative to separated reacting molecules) for both the isolated and SCRF model are also displayed.

In step (1) singlet spin state is clearly disfavored; however, energy difference between the triplet and singlet state gradually decreases on abstraction of hydrogen at either side. Polar medium further enhances this trend, since the polarity of the system steadily increases during step (1). Energies of the corresponding entities in the singlet state are displayed in S5 (Figs. S18-19) where the successive steps (1) and (5) are presented as a whole for practical reasons.

S2. Details on step (2): $LFNH_2$ + HOO• → LFNH• + H_2O_2

This step features little if any relevance for the overall mechanism and kinetics, because step (1) prevails over it regardless of the barrier. Namely, if step (2) is of a very low barrier or even barrierless-downhill, then the required energy input of precedent step (1) represents the effective barrier (see Figs. S1-2 and Table 1 in the main text). On the other hand, if the barrier pertinent to step (2) is higher than the required energy input for initialization of radicals in step (1), then step (2) will be kinetically disfavored to step (1) and will likely represent statistically irrelevant part of the reaction pathway. Consequently, in-depth elucidation of step (2) is not required, but certain aspects are worthy to note.

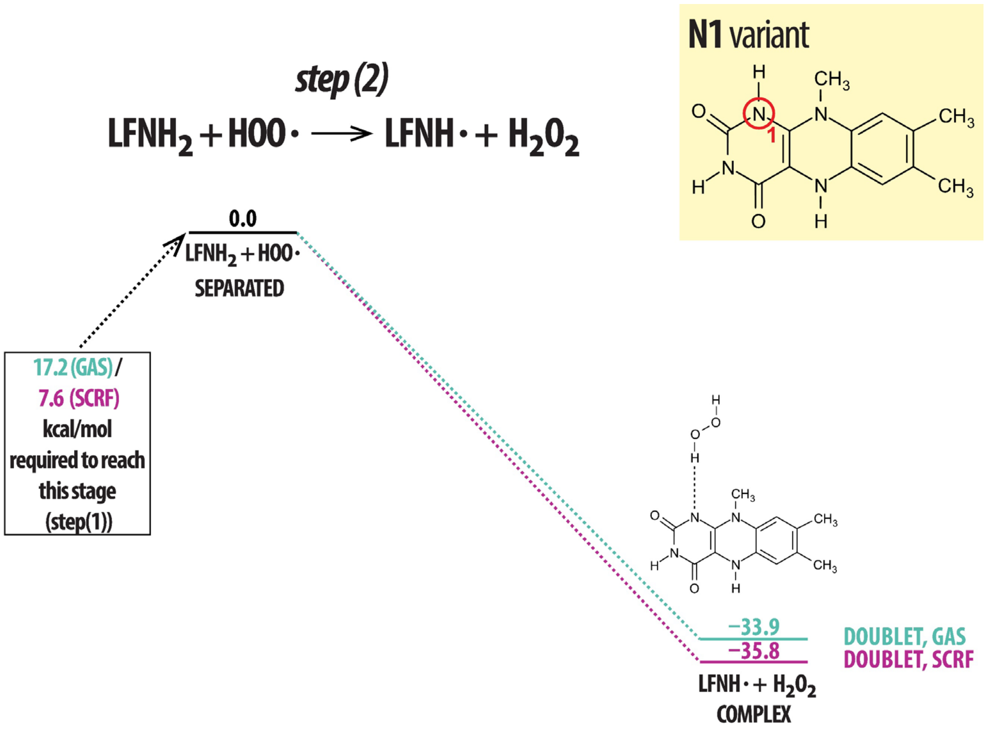

Figure S5. Schematic representation of step (2) of oxidation of flavin (*N*1 variant). Color code: green – triplet spin state in the gas phase; purple – triplet spin state, implicit solvation model (SCRF; water as solvent). All energies are given in kcal/mol relative to the separated reactant molecules in the respective phase (gas or SCRF). Energy cost to acquire the reacting species in the initialization step (step 1) is also given for both models.

Energetics of step (2) is schematically presented in Fig. S5 and S6 for the *N*1 and *N*5 abstraction variant, respectively, whereas geometric features of the corresponding stationary points of the *N*5 variant are displayed in Fig. S7. All the involved entities of step (2) are evidently in the spin doublet state.

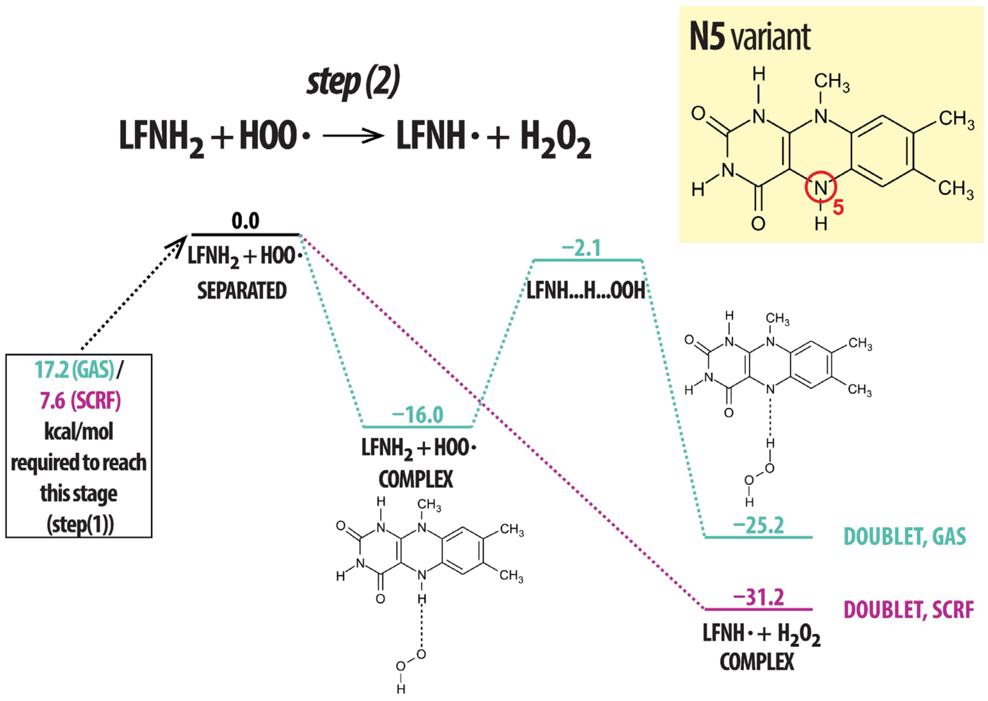

Figure S6. Schematic representation of step (2) of oxidation of flavin (*N*5 variant). Color code and energy definition are the same as in Fig. S5.

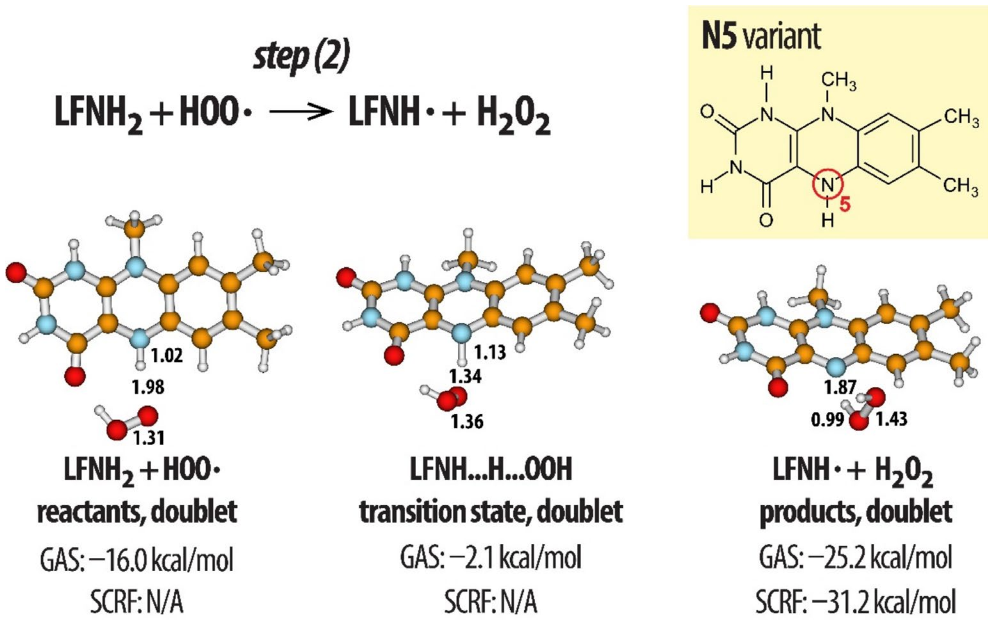

Figure S7. Characteristic structures involved in step (2) of oxidation of flavin (*N*5 variant) together with N…H, H…O and O…O distances (in Å). Energies of entities (relative to separated reacting molecules) for both the isolated and SCRF model are also displayed.

With the exception of hydrogen abstraction at *N*5 in the gas phase the reaction appears to proceed spontaneously – the system relaxes into products, and attempts at finding a stable reactant complex fail. This suggests that hydrogen transfer occurs in a barrierless-downhill fashion, the product complex being at –33.9 kcal/mol relative to separated reactants for abstraction at N1 in the gas phase (Fig. S5). For reaction in implicit

solvent the equivalent energy values of product complex are –35.8 and –31.2 kcal/mol for N1 and N5 abstraction, respectively (Figs. S5-6).

On the other hand, hydrogen abstraction from *N*5 features a stable, quite strongly bound complex at –16.0 kcal/mol kcal/mol relative to separated $LFNH_2$ and HOO• molecules, followed by a transition state at –2.1 kcal/mol, then relaxing to LFNH•…H_2O_2 product complex at –25.2 kcal/mol. Geometry of the reactant complex confirms a somewhat stronger interaction than in step (1), in that the O…N separation of ~2.0 Å is shorter. In the transition state characteristic effects of hydrogen transfer in hydrogen bonds are observed, with strongly reduced N…O separation and the hydrogen atom shifted towards the midpoint.

All in all, for all presently employed models of step (2), we found that the highest point on the reaction profile (if any) does not exceed the energy of separated reactants previously supplied by step (1), therefore step (2) does not have an impact on reaction kinetics. The fact that gas phase reaction at *N*5 proceeds *via* a stable complex and transition state implicitly confirms what has been observed at step (1), namely that hydrogen abstraction from *N*5 is less favorable in comparison with *N*1.

S3. Details on step (3): LFNH• + $O_2 \rightarrow$ LFN + HOO•

Energy profiles of step (3) are schematically presented in Fig. S8 and S9 for the *N*1 and *N*5 abstraction variant, respectively. Geometries of the characteristic entities of both variants in the gas phase are shown in Fig. S10 and Fig. S11, whereas those pertaining to aqueous solution are displayed in Fig. S12 and S13.

As mentioned in the main text, this step features a change in the spin state of the system from initial quartet (corresponding to non-interacting LFNH• and O_2 molecules that exist in a doublet and triplet state, respectively) to doublet products consisting of the spin-paired LFN molecule and the HOO• radical. A notable difference between the gas-phase and implicit solvation model has been observed, in that in the gas phase the quartet-to-doublet transition occurs at the stage of the reacting complex, as confirmed by MECP optimization, whereas in solution the MECP occurs somewhat later, with the N…O and H…O distances substantially shortened, but with hydrogen still firmly at the donor (flavin) site (Figs. S12-13).

In all cases the LFNH• and O_2 molecules form a weakly bound complex stabilized by 1.5-3.3 kcal/mol depending on the abstraction site and the environment (Figs. S8-9); geometries of the N—H…O moiety confirm a weak interaction with N…O separation of 2.2-2.3 Å (Figs. S10-11). In the gas phase hydrogen transfer from LFNH• to O_2 proceeds via a compact transition state with short donor-acceptor separation of less than 2.5 Å (Figs. S10-11), which is essentially equivalent to proton transfer in hydrogen bonds. Further along the reaction course the energy of the quartet state is persistently higher than that of the doublet state. As the transition between the two states occurs already at the level of reactants, we only consider the doublet reaction profile in the gas phase (displayed in green in Figs. S8-9). Here, the *N*5 variant appears to be significantly preferred over *N*1, in that it features by ~13 kcal/mol lower barrier and by ~10 kcal/mol higher exergonicity. This is in accordance with the perceivably easier hydrogen abstraction from the *N*1 site observed in step (1) (please note that in the *N*5 variant of this step hydrogen abstraction takes place at the *N*1 site and *vice versa* in the *N*1 variant). However, in large part the preference of the *N*5 variant is compensated by the larger energy cost of obtaining LFNH• radicals from initial reactants within step (1), which is by ~9 kcal/mol less favorable for the *N*5 variant.

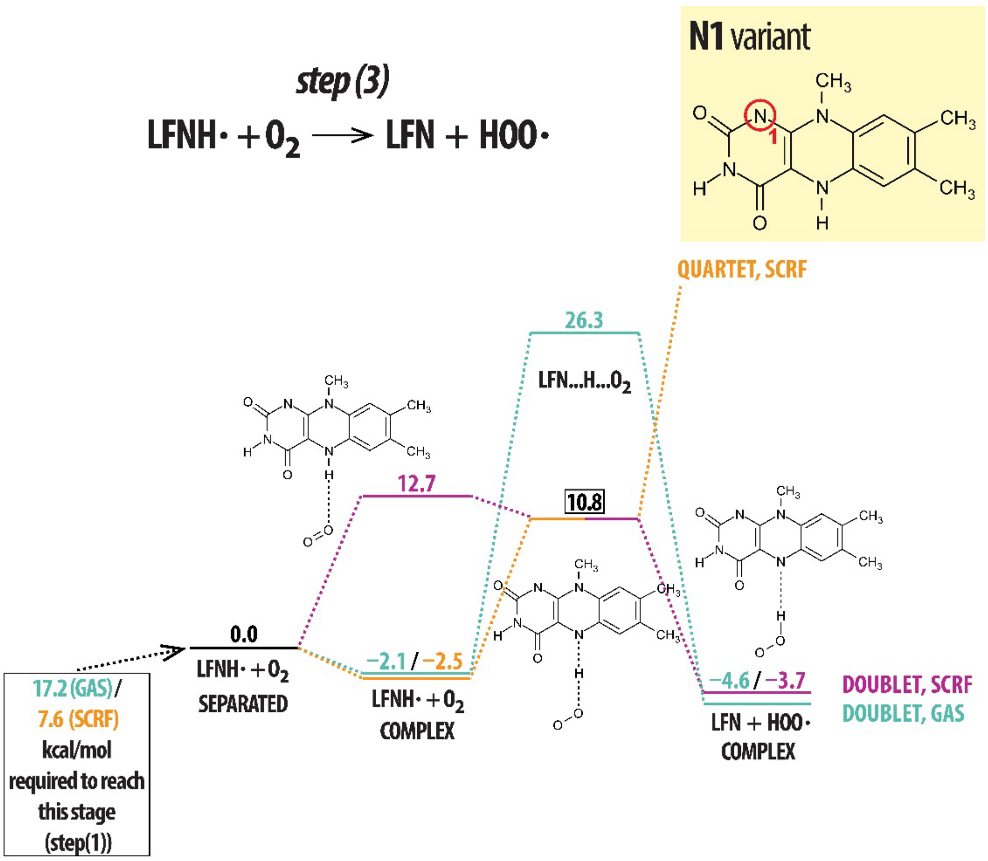

Figure S8. Schematic representation of step (3) of oxidation of flavin (*N*1 variant). Color code: purple – doublet spin state in the gas phase; green – doublet spin state, implicit solvation model (SCRF; water as solvent); orange: quartet spin state, SCRF. All energies are given in kcal/mol relative to the separated reactant molecules in the respective phase (gas or SCRF). The framed value of 10.8 kcal/mol denotes the minimum energy crossing point (MECP) between the quartet and doublet state. In bottom left is indicated the energy cost of obtaining the reacting entities from initial LFNH$_2$ and H$_2$O$_2$ reactants in step (1). Please note that the *N*1 notation corresponds to the site from which the first hydrogen has been abstracted; since abstraction of first hydrogen occurs in step (1), the remaining hydrogen is abstracted from *N*5 site in the *N*1 variant (and *vice versa* for the *N*5 variant).

In contrast to the gas phase, the intersection between the quartet and doublet spin state in the solution occurs later in the reaction course; for the reactant complex, the quartet state is by ~12 kcal/mol more favorable than doublet. The optimized MECP of the quartet and doublet surface (Figs. S12-13) is estimated to 10.8 kcal/mol for the *N*1 variant, and substantially less, only 1.3 kcal/mol for the *N*5 variant, respectively (Figs. S8-9), confirming clear kinetic preference of the latter. No transition state could be found on the doublet surface, and the structure of the doublet state is spontaneously relaxed to products after passing MECP. Therefore, the quartet-doublet MECP represents the highest point on the SCRF reaction path and energy of MECP represents the effective barrier of step (3).

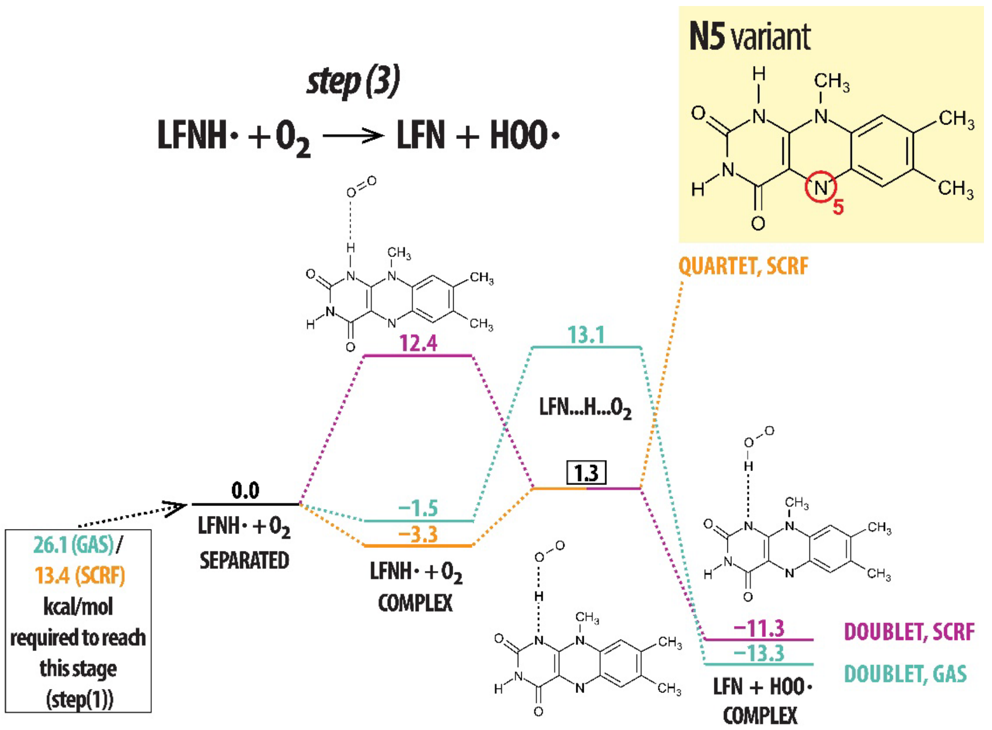

Figure S9. Schematic representation of step (3) of oxidation of flavin (*N*5 variant). Color code and energy definitions are the same as in Fig. S8.

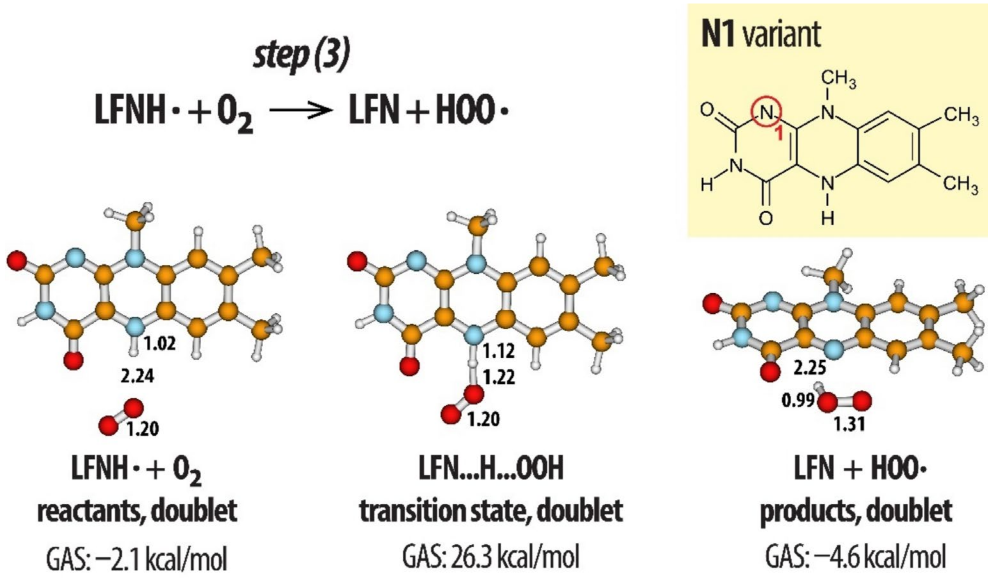

Figure S10. Characteristic structures involved in step (3) of oxidation of flavin (*N*1 variant, gas phase) together with N…H, H…O and O…O distances (in Å). Energies of entities (relative to separated reacting molecules) are also displayed.

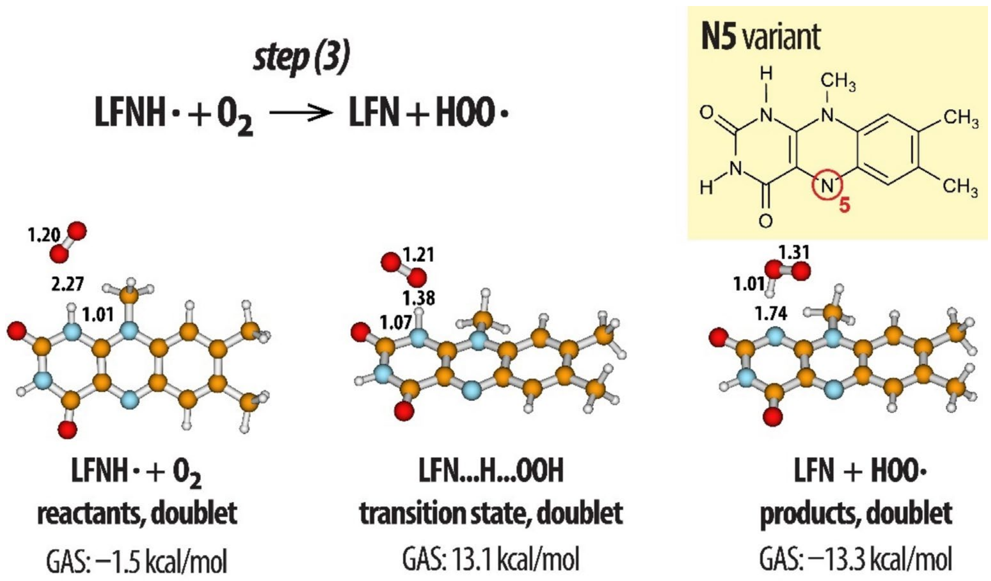

Figure S11. Characteristic structures involved in step (3) of oxidation of flavin (*N*5 variant, gas phase) together with N…H, H…O and O…O distances (in Å). Energies of entities (relative to separated reacting molecules) are also displayed.

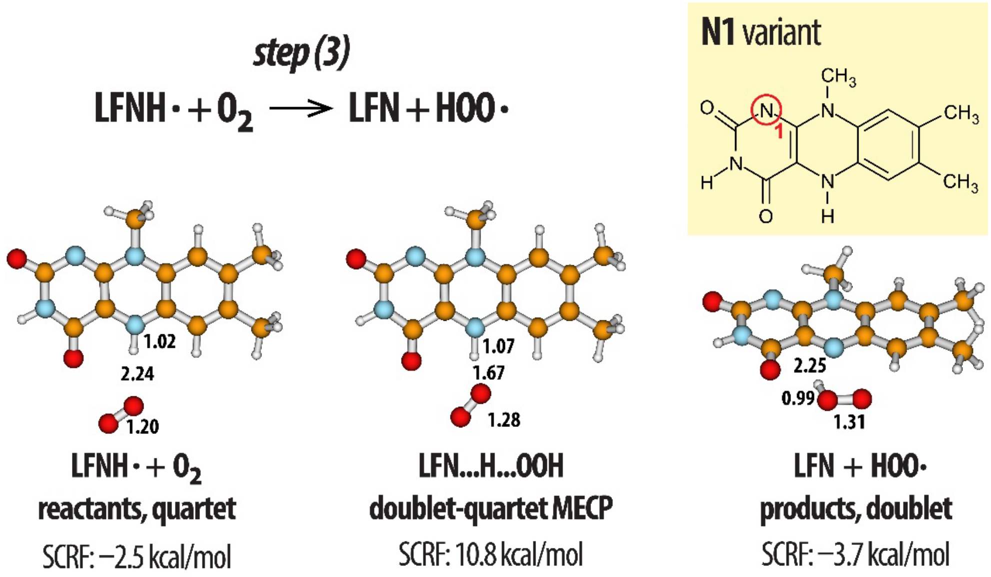

Figure S12. Characteristic structures involved in step (3) of oxidation of flavin (*N*1 variant, implicit solvation (SCRF) model) together with N…H, H…O and O…O distances (in Å). Energies of entities (relative to separated reacting molecules) are also displayed.

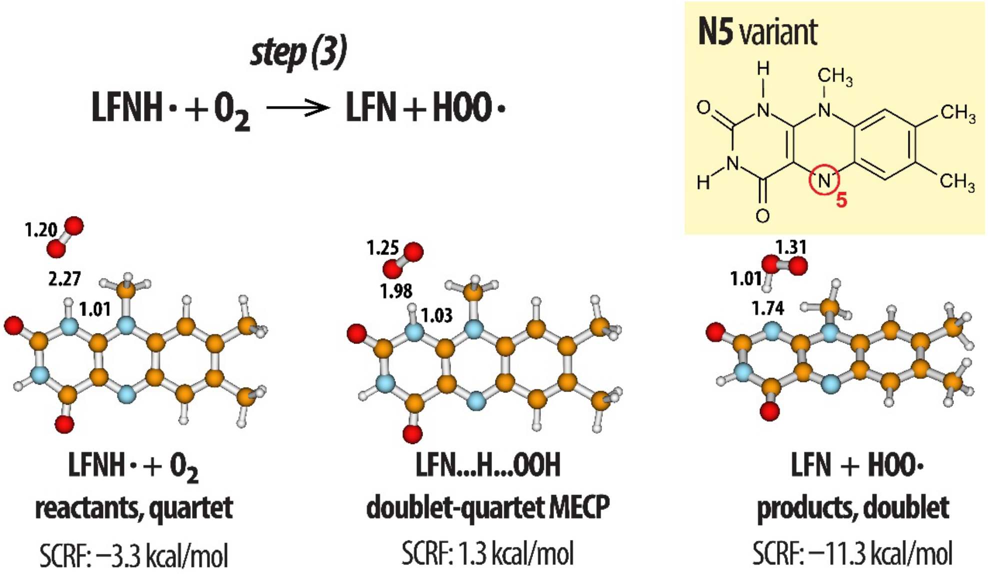

Figure S13. Characteristic structures involved in step (3) of oxidation of flavin (*N*5 variant, implicit solvation (SCRF) model) together with N...H, H...O and O...O distances (in Å). Energies of entities (relative to separated reacting molecules) are also displayed.

S4. Details on step (4): LFNH• + LFNH• → LFN + $LFNH_2$

Similarly to step (3), a change of spin (from triplet to singlet) occurs during the reaction, with triplet state being prevalent over singlet by ~30 kcal/mol at the reactant side in both media. The triplet reaction profiles appear to be of barrierless-uphill type whereas for the singlet state they are just the opposite. The assumed minimum energy path (Fig. S4b) includes the uphill triplet state potential which switches to the singlet potential at their intersection. From that point on, relaxation to products is of a barrierless-downhill type. Therefore, the MECP between the respective potential energy surfaces represents the estimate of the effective barrier for this step.

Table S1. Energies of characteristic structures involved in the LFNH• + LFNH• → LFN + $LFNH_2$ reaction (step (4)) computed for the four possible tautomeric forms, as well as for an isolated (gas) and implicit solvation (SCRF) model: (i) reactant complex in the triplet state; (ii) the minimum energy crossing point (MECP) between the triplet and singlet potential energy surface; (iii) the product complex in the singlet state. These stages are part of the minimum energy path of the reaction accompanied by the spin change from triplet to singlet. Entry (iv) includes the energy cost for the formation of two LFNH• radicals produced in step (1), whereas (v) is the total barrier estimate of successive steps (1) and (4), which is the sum of terms in (ii) and (iv). Entry (vi) is the reaction energy of successive steps (1) and (4), obtained by summing of (iii) and (iv). All values are given in kcal/mol.

variant (tautomer)		energy relative to separated reactants of step (4) (two LFNH• molecules)						energy relative to separated reactants of step (1) ($LFNH_2$ and O_2)					
H donor	H acceptor	(i) reactant complex (triplet)		(ii) triplet-singlet MECP		(iii) product complex (singlet)		(iv) cost to form 2 LFN• radicals (step (1)/Table 1)		(v) total barrier (MECP + step 1)		(vi) total energy (product complex + step 1)	
		gas	SCRF	gas	SCRF	gas	SCRF	gas	SCRF	gas	SCRF	gas	SCRF
***N*1**	***N*1**	−16.1	−11.0	−11.9	−6.0	−33.0	−18.1	43.3	22.0	31.4	16.0	10.3	3.9
***N*1**	***N*5**	−21.0	−7.8	−10.6	−3.7*	−43.9	−30.0	52.2	26.8	41.6	23.1*	8.3	−3.2
***N*5**	***N*1**	−27.3	−14.0	−13.5	−8.5	−37.8	−22.9	34.4	15.2	20.9	6.7	−3.4	−7.7
***N*5**	***N*5**	−19.1	−7.0	−6.8	0.9*	−35.1	−23.4	43.3	22.0	36.5	22.9*	8.2	−1.4

*** SCRF MECP optimization of (donor/acceptor) N1/N5 and N5/N5 variants did not succeed, non-optimized evaluations derived from precedent potential energy surface scans are given instead.**

Another feature requiring consideration is the isomerism of the LFNH• molecule (see Fig. 4), yielding four possible tautomeric forms of the LFNH•...LFNH• complex (see Fig. S14), each having slightly different reaction energy and MECP/barrier. These quantities are listed in Table S1 for each of the four tautomers. Energetics of

the preferred variant of the reaction (with *N*5 as hydrogen donor and *N*1 as hydrogen acceptor) is schematically presented in Fig. S15. Geometric features of the involved entities in both media are displayed in Figs. S16-17.

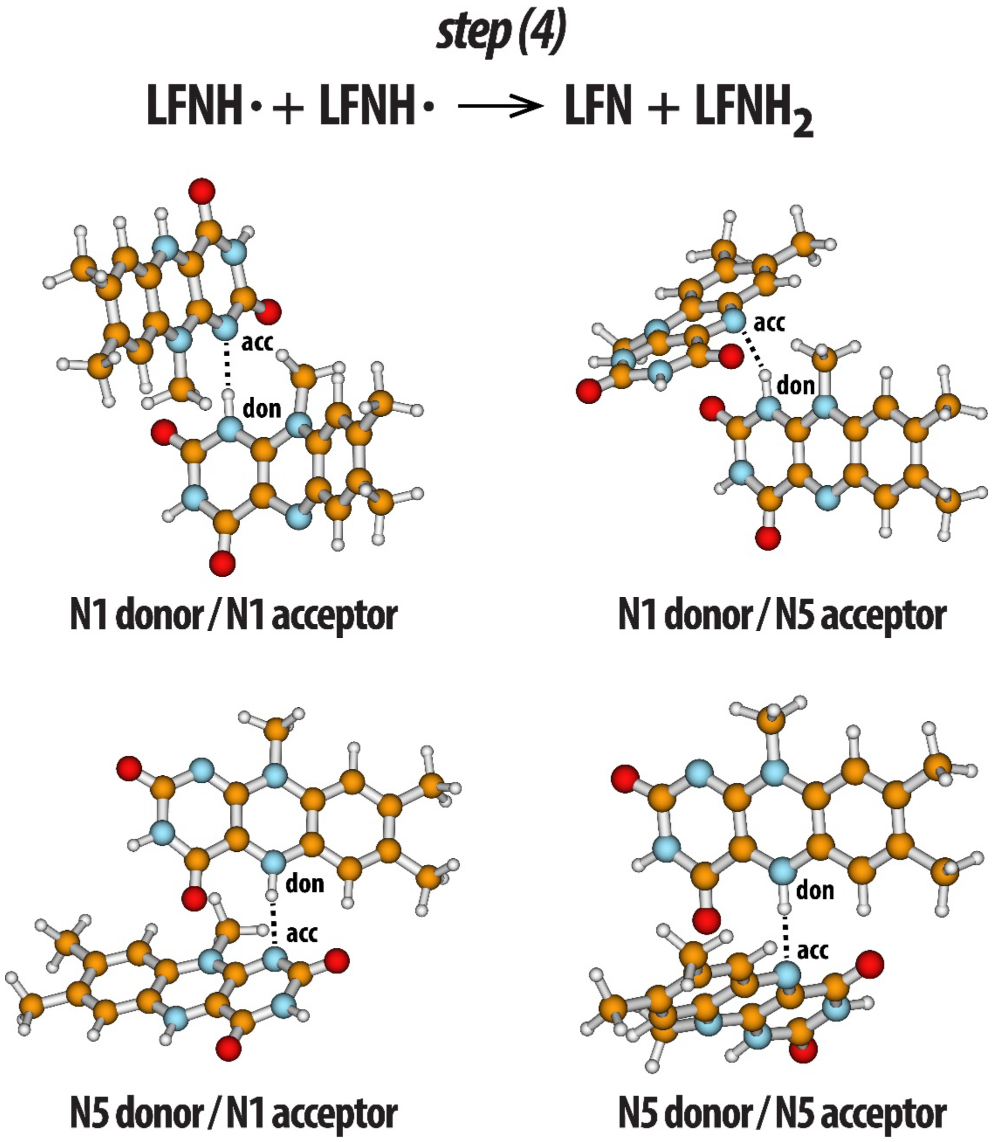

Figure S14. Tautomers of the reacting complex of two LFNH• molecules with hydrogen donor and acceptor atom indicated.

While relatively large variations in the barrier and reaction energy exist among the tautomers, gas phase barriers for the reaction consisting of successive steps (1) and (4) are perceivably higher (between 20.9 and 41.6 kcal/mol) than in solution (between 6.7 and 23.1 kcal/mol) (columns (v) in Table S4). Considering the most favorable among those, the estimated barriers are just above 20 kcal/mol in the gas phase and only 6.7 kcal/mol in the solution. At the same time, the total reaction is slightly exergonic (–3.4 and –7.7 kcal/mol for the gas phase and implicit solvation, respectively). Therefore, step (4) appears to be both kinetically and thermodynamically favored over step (3) in which the barriers and reaction energies are larger by 8-18 kcal/mol. The computed lowest barrier in the solution (6.7 kcal/mol) is substantially lower than the experimentally deduced barrier in the active site of MAO-B. However, the fact that step (4) requires interaction of a pair of LFNH• entities speaks strongly against feasibility of such mechanism within an enzyme, because the active sites of flavoenzymes typically contain only one flavin entity (covalently bound to the enzyme scaffold, as for example in MAO enzymes). Involvement of two flavin molecules in the active site would require both (temporary) disintegration of the active site of an enzyme

by protein unfolding, as well as the existence of two flavin molecules in the active site of an enzyme, which is far from being a reasonable assumption. Namely, enzyme unfolding is associated with too high free energy cost, rendering this mechanism highly unlikely. Therefore, one may assume another plausible flavin oxidation mechanism may be in operation.

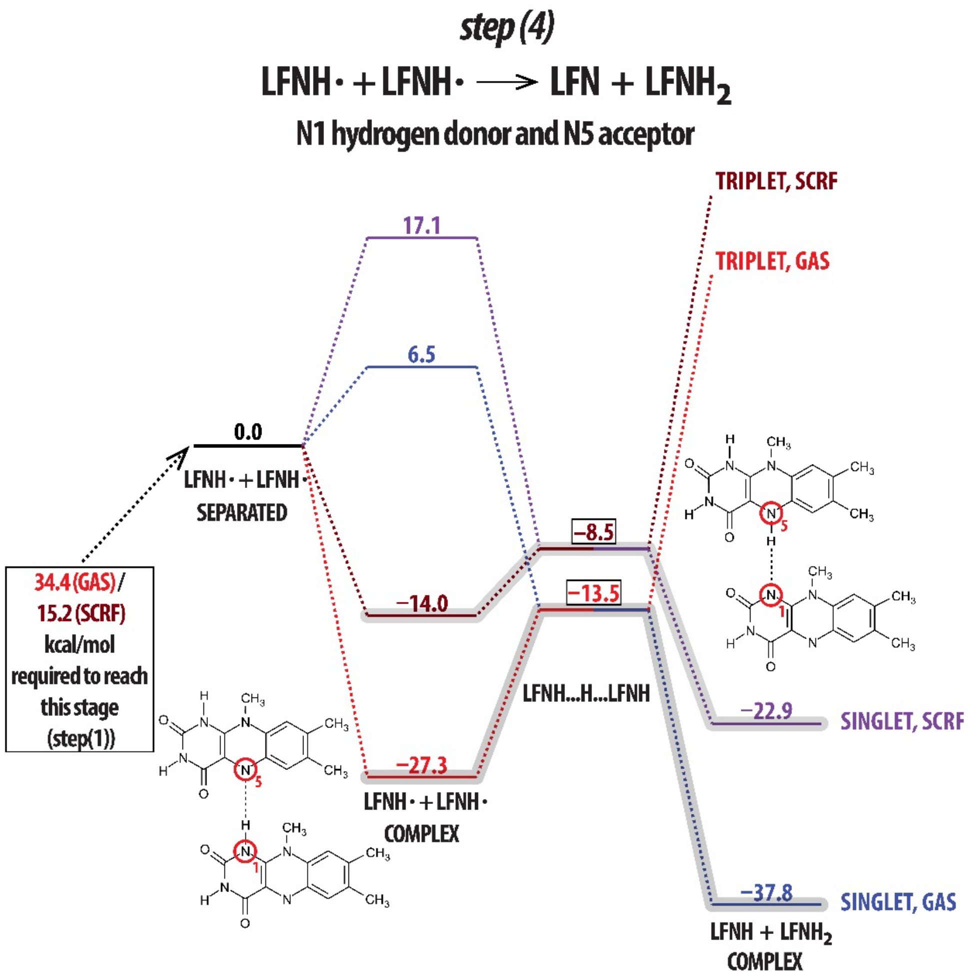

Figure S15. Schematic representation of step (4) of oxidation of flavin (the variant with *N*1 as hydrogen donor and *N*5 as acceptor, which is the favored variant among possible isomers of an adduct comprised by two LFNH• molecules). Color code: red – triplet spin state in the gas phase; brown – triplet spin state, implicit solvation model (SCRF; water as solvent); blue: singlet spin state, gas phase; purple: singlet spin state, SCRF. All energies are given in kcal/mol relative to the separated reactant molecules in the respective phase (gas or SCRF). The framed values of –8.5 and –13.5 kcal/mol denote the minimum energy crossing point (MECP) between the triplet and singlet potential surface. At left is indicated the energy cost of obtaining the reacting entities (two LFNH• molecules) from initial $LFNH_2$ and H_2O_2 reactants in step (1). The minimum energy path combining both spin states is outlined in light grey for both the gas phase as well as for the solution model.

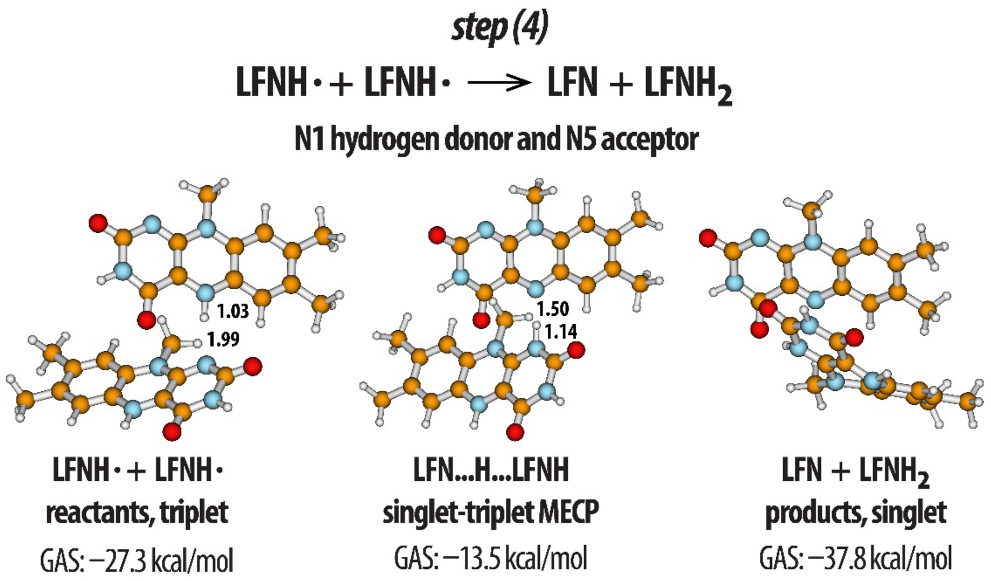

Figure S16. Characteristic structures involved in step (4) of oxidation of flavin for the most favorable variant (*N*1 as H-donor and *N*5 as H-acceptor, see Fig. S14) together with N…H, H…O and O…O distances (in Å) computed for a gas phase model. Energies of entities (relative to separated reacting molecules) for the isolated model are also displayed.

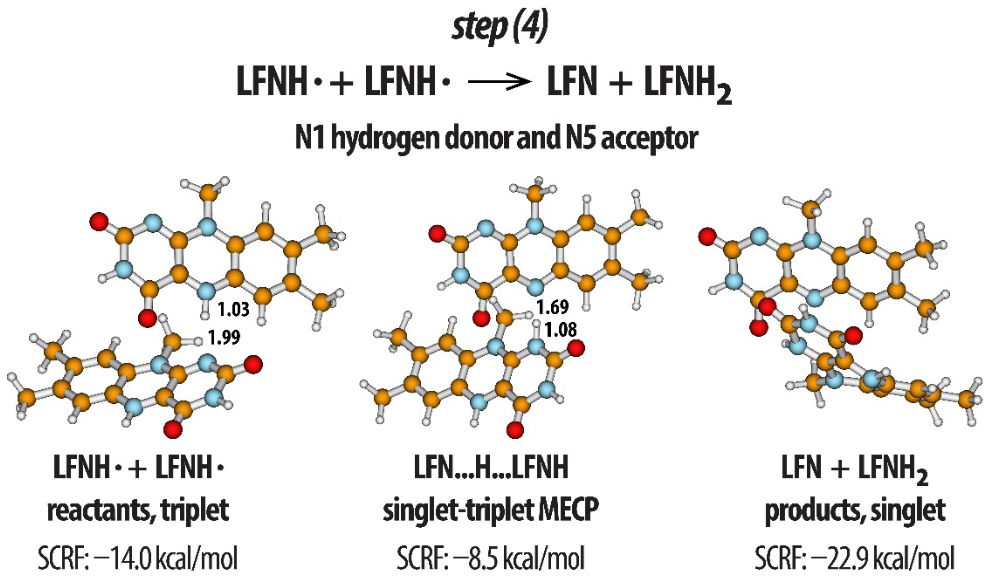

Figure S17. Characteristic structures involved in step (4) of oxidation of flavin for the most favorable variant (*N*1 as H-donor and *N*5 as H-acceptor, see Fig. S14) together with N…H, H…O and O…O distances (in Å) computed for an implicit solvent (SCRF) model. Energies of entities (relative to separated reacting molecules) for the isolated model are also displayed.

S5. Details on step (5): LFNH• + HOO• → LFN + H_2O_2

Unlike profiles of steps (2), (3) and (4), no correction for the cost of formation of reacting entities is required for step (5) because step (5) directly uses both radical species produced in step (1). For the same reason there is no need to consider dissociation of products acquired in step (1). Therefore, the overall reaction consisting of successive steps (1) and (5) can be treated and displayed as a whole. While the *N*1 variant of the overall reaction exhibiting kinetic preference – quite sizable for the gas phase but quite small for the aqueous solution – is schematically presented in the main text (Fig. 5), the *N*5 variant is presented in Fig. S18. Figs. S19-22 show characteristic structures of the reaction (reactants, triplet-singlet MECP and products) for the *N*1 and *N*5 variant, respectively, and their selected geometry features in both types of media.

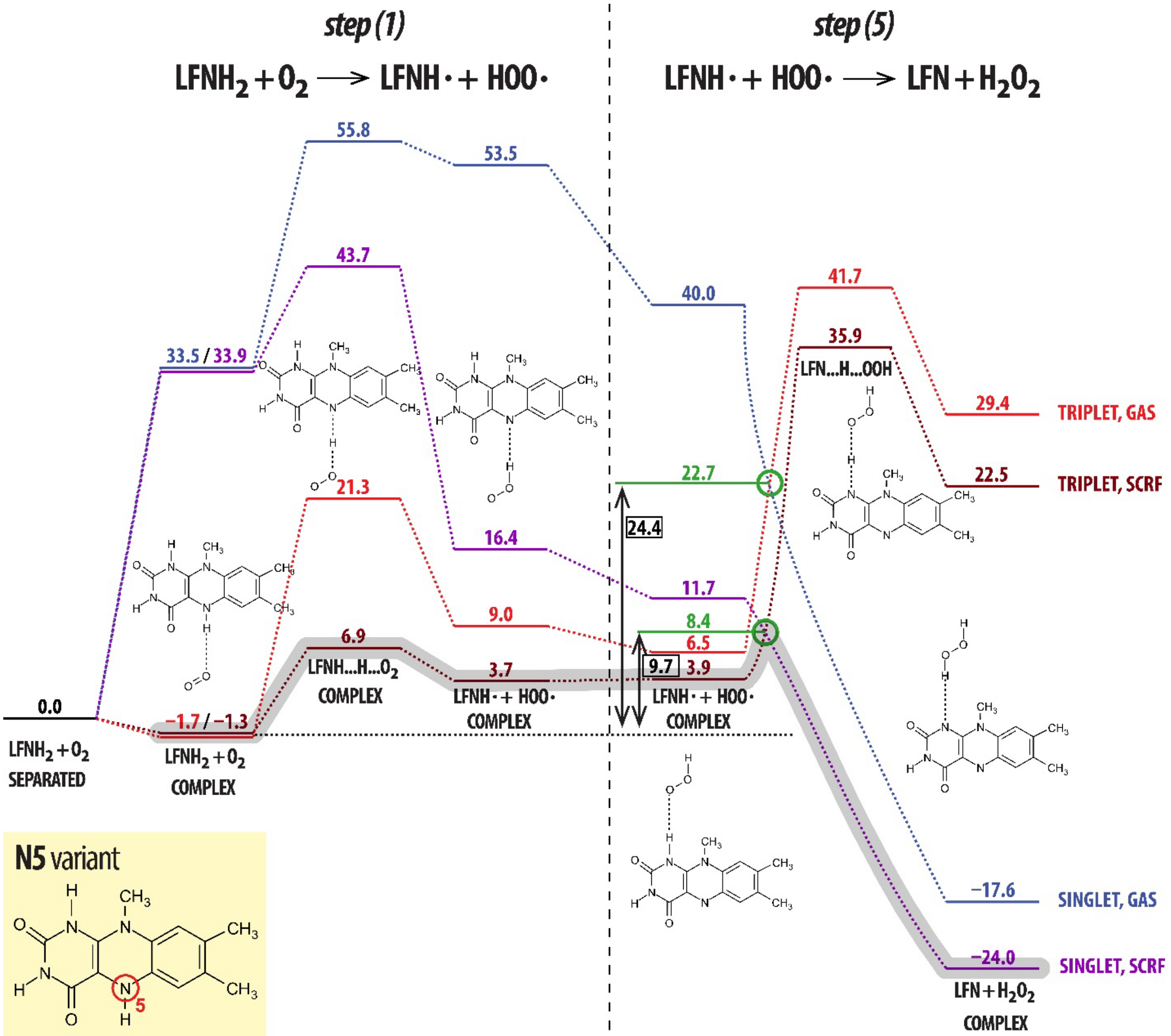

Figure S18. Schematic representation of oxidation of flavin proceeding by a radical mechanism (*N*5 variant) consisting of steps (1) and (5). Color code: red – triplet spin state in the gas phase; brown – triplet spin state, implicit solvation model (SCRF; water as solvent); blue – singlet spin state in the gas phase; purple – singlet spin state, implicit solvation model. The minimum energy crossing point (MECP) between triplet and singlet state is shown in green for both phases. All displayed values are energies (in kcal/mol) of the corresponding species given relative to the isolated reactants in their ground state, except for the framed black values which correspond to the effective barrier in the gas phase and in the solvent reaction field (given relative to the $LFNH_2 \ldots O_2$ reactant complex). The minimum energy path combining both spin states is outlined in light grey for the solution model.

The reactant complex of LFNH• and HOO• is the same as the one formed in step (1), but rearranged between *N*1 and *N*5 sites as to ensure abstraction of the remaining hydrogen atom bound either to *N*1 or *N*5 ring nitrogen. For both variants the reactant complex features a moderate N—H…O(OH) hydrogen bond which is, at ~2.8 Å N…O separation (Fig. S19-22), just slightly shorter than one in the reactant complex of step (2), but substantially shorter than ones formed between flavin and molecular oxygen in steps (1) and (3). At the reactant stage, triplet spin state is favored by more than 35 kcal/mol in the gas phase, but much less so, 20 kcal/mol at worst, in the solution.

Regardless of the variant, the triplet reaction profile features a regular transition state and products, however, the reaction is highly endergonic and the barrier is high. Depending on the variant, the barrier is at least 35 kcal/mol above the reactant complex and the products are more than 22 kcal/mol above the reactants at best

(Fig. 5 in the main text and Fig. S18). Solvent effects render the triplet profiles slightly less unfavorable, but the barrier is still at least 28 kcal/mol above reactants, and endergonicity amounts to at least 18 kcal/mol. In contrast to the uphill triplet profile, the singlet surface features a virtually barrierless relaxation to products both in the gas phase and in solution, and no stable reactant minimum could be obtained by optimization in the singlet state. MECP optimization in the gas phase yields for the *N*1 variant a transition-state-like structure with very short O…O separation of 2.47 Å and the hydrogen atom near the N…O midpoint (Fig. S19), whereas MECP of the *N*5 variant looks more like reactant complex with hydrogen firmly at the donor (flavin) site and the relatively large N…O separation of 2.75 Å (Fig. S20). In the polar solvent, the optimized MECP's of both variants are similar to those obtained in the gas phase, but with longer donor-acceptor separation of 2.68 and 2.90 Å for the *N*1 and *N*5 variant, respectively (Figs. S21-22). As has been presented in the main text, the computed MECP's of step (5) are perceivably lower in energy than in step (3) or step (4), so that for the *N*1 variant the effective barrier of the net reaction consisting of consecutive steps (1) and (5) is attributed to the transition state of step (1). In contrast, MECP remains at the highest point of the profile for the *N*5 variant, but barely higher in energy than the transition state of step (1). After switching to the singlet surface, substantial relaxation to products follows, rendering the entire reaction strongly exergonic.

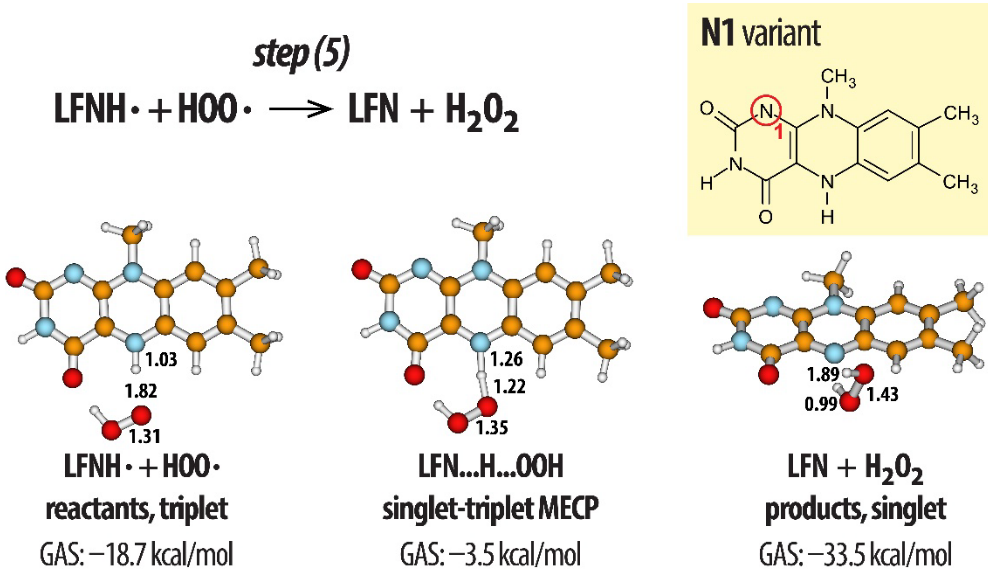

Figure S19. Characteristic structures involved in step 5 of oxidation of flavin (*N*1 variant, gas phase model) together with N…H, H…O and O…O distances (in Å). Energies of entities (relative to separated reacting molecules) are also displayed.

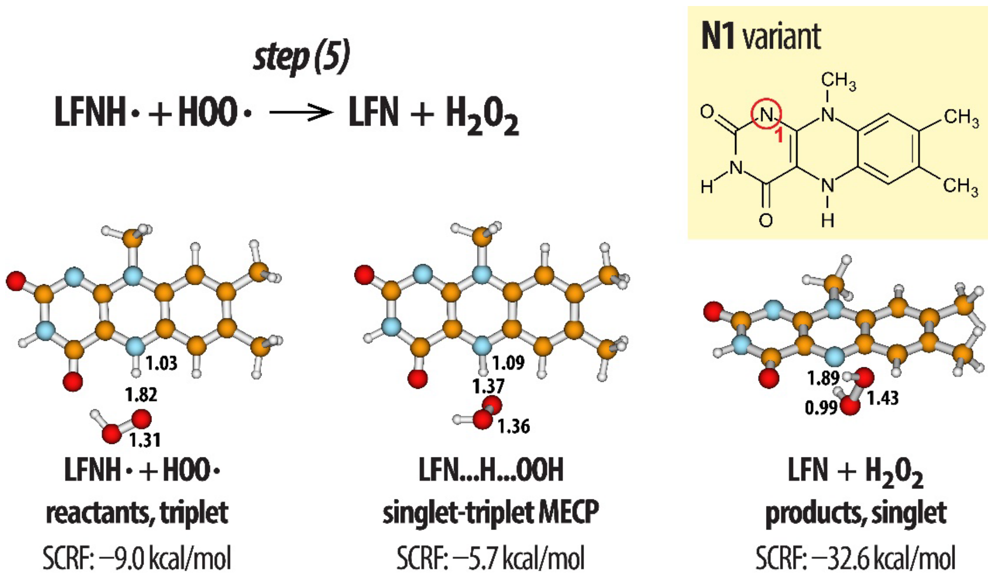

Figure S20. Characteristic structures involved in step 5 of oxidation of flavin (*N*1 variant, SCRF model) together with N…H, H…O and O…O distances (in Å). Energies of entities (relative to separated reacting molecules) are also displayed.

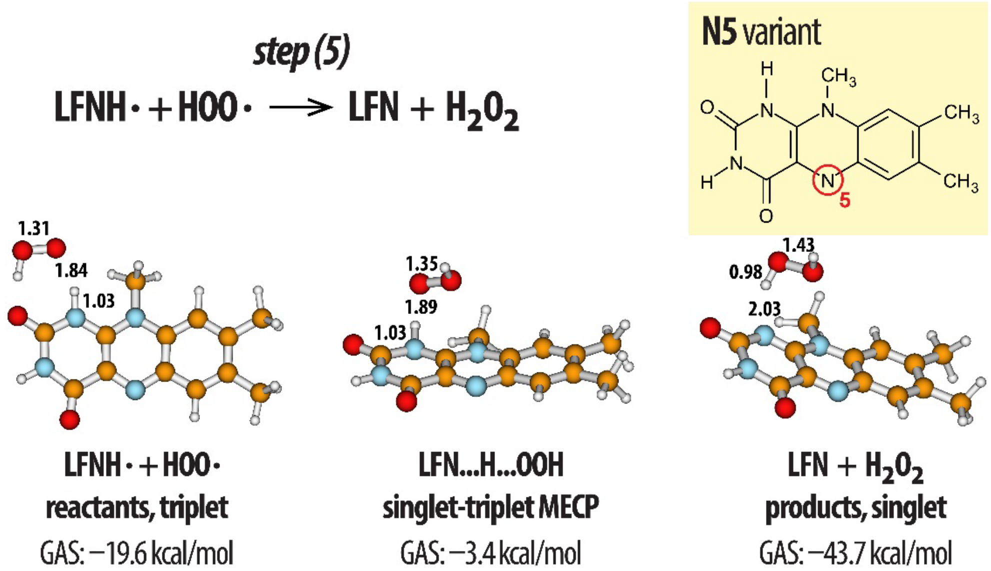

Figure S21. Characteristic structures involved in step 5 of oxidation of flavin (*N*5 variant, gas phase model) together with N…H, H…O and O…O distances (in Å). Energies of entities (relative to separated reacting molecules) are also displayed.

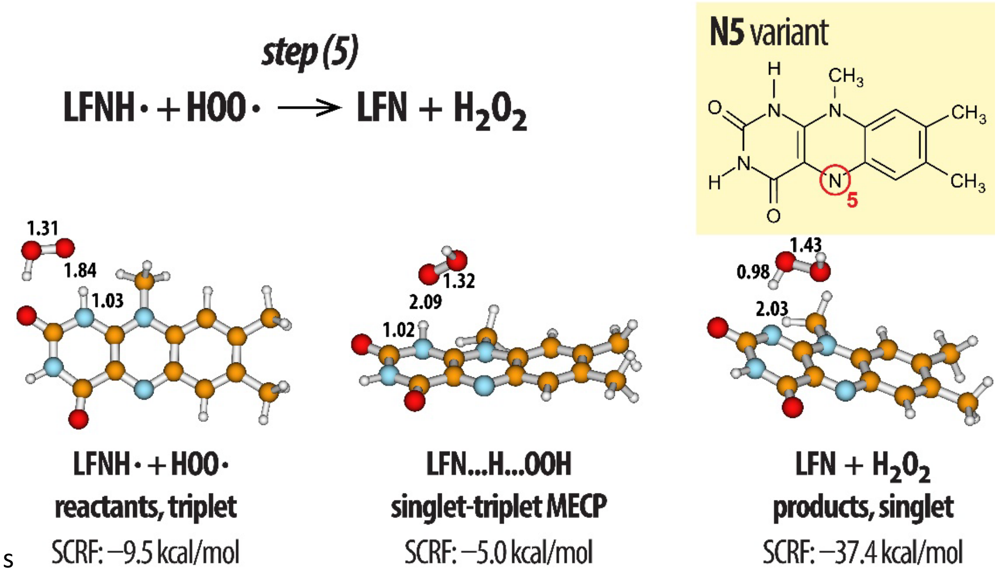

Figure S22. Characteristic structures involved in step 5 of oxidation of flavin (*N*5 variant, SCRF model) together with N…H, H…O and O…O distances (in Å). Energies of entities (relative to separated reacting molecules) are also displayed.

S6. Benchmark calculations / comparison of different functionals

As mentioned in Computational Details, we benchmarked the herein employed M06-2X functional against three other popular functionals, namely B3LYP, BYLP and PBE, by optimizing the following three characteristic points on the reaction profile: (i) reactant complex of step (1); (ii) transition state of step (1); and (iii) triplet-singlet minimum energy crossing point of step (5). These entities and their energies appear to be crucial for the kinetics of the reaction (see Fig. 5 of the main text and Fig. S18). The same 6-31+G(d,p) basis set was used alongside with the functionals. Table S2 lists the computed energies relative to the optimized reactants of step (1).

Table S2. Relative energies of selected characteristic entities computed by optimization using different functionals together with the 6-31+G(d,p) basis set, namely: R – reactant complex of step (1); TS – transition state of step (1); 3-1 MECP – triplet-singlet minimum energy crossing point of step (5). The *N*1 variant of the mechanism has been assumed and an isolated model was used. All values are given in kcal/mol.

entity/DFT	PBE	BLYP	B3LYP	M06-2X
R	0.0	0.0	0.0	0.0
TS	6.4	7.2	9.7	16.6
3-1 MECP	1.7	1.4	7.2	15.2

The M06-2X functional yields substantially higher barrier of step (1) and also higher 3-1 MECP of step (5) than all other functionals. In this regard, the M06-2X functional appears to be quite 'different' from the other three, yielding by ~7, 9, and 10 kcal/mol higher barriers than B3LYP, BLYP and PBE functionals, respectively. On the other side, performance analysis of B3LYP, BLYP and PBE functionals demonstrated that these functionals considerably underestimate hydrogen transfer reaction barriers – on the average by approximately 4.1, 7.5 and 9.3 kcal/mol, respectively, against CCSD(T) reference [Y. Zhao, N. González-García, *J. Phys. Chem. A* **2005**, *109*, 2012.]. Taking into account this fact, the herein observed discrepancy between M06-2X and other functionals suggests that the latter may eventually be quite accurate for the reaction in question, thereby validating the present approach. A more thorough validation would include benchmarking against a superior post-SCF approach (e. g., CCSD(T)), but for the present system such calculations are prohibitively expensive. All in all, the herein found trend among functionals confirms the M06-2X as a reasonable choice.
